# The Impact of Sparse Coding on Memory Lifetimes in Simple and Complex Models of Synaptic Plasticity

**DOI:** 10.1007/s00422-022-00923-y

**Published:** 2022-03-14

**Authors:** Terry Elliott

**Affiliations:** Department of Electronics and Computer Science, University of Southampton, Highfield, Southampton, SO17 1BJ UK

**Keywords:** Synaptic plasticity, Memory models, Sparse coding, Stochastic processes

## Abstract

Models of associative memory with discrete state synapses learn new memories by forgetting old ones. In the simplest models, memories are forgotten exponentially quickly. Sparse population coding ameliorates this problem, as do complex models of synaptic plasticity that posit internal synaptic states, giving rise to synaptic metaplasticity. We examine memory lifetimes in both simple and complex models of synaptic plasticity with sparse coding. We consider our own integrative, filter-based model of synaptic plasticity, and examine the cascade and serial synapse models for comparison. We explore memory lifetimes at both the single-neuron and the population level, allowing for spontaneous activity. Memory lifetimes are defined using either a signal-to-noise ratio (SNR) approach or a first passage time (FPT) method, although we use the latter only for simple models at the single-neuron level. All studied models exhibit a decrease in the optimal single-neuron SNR memory lifetime, optimised with respect to sparseness, as the probability of synaptic updates decreases or, equivalently, as synaptic complexity increases. This holds regardless of spontaneous activity levels. In contrast, at the population level, even a low but nonzero level of spontaneous activity is critical in facilitating an increase in optimal SNR memory lifetimes with increasing synaptic complexity, but only in filter and serial models. However, SNR memory lifetimes are valid only in an asymptotic regime in which a mean field approximation is valid. By considering FPT memory lifetimes, we find that this asymptotic regime is not satisfied for very sparse coding, violating the conditions for the optimisation of single-perceptron SNR memory lifetimes with respect to sparseness. Similar violations are also expected for complex models of synaptic plasticity.

## Introduction

One line of experimental evidence suggests that synapses may occupy only a very limited number of discrete states of synaptic strength (Petersen et al. 1998; Montgomery and Madison 2002, 2004; O’Connor et al. 2005a, b; Bartol et al. 2015), or may change their strengths via discrete, jump-like processes (Yasuda et al. 2003; Bagal et al. 2005; Sobczyk and Svoboda 2007). Discrete state synapses overcome the catastrophic forgetting of the Hopfield model (Hopfield 1982) in associative memory tasks, turning memory systems into so-called palimpsests, which learn new memories by forgetting old ones (Nadal et al. 1986; Parisi 1986). Unfortunately, memory lifetimes in the simplest such models are rather limited, growing only logarithmically with the number of synapses (Tsodyks 1990; Amit and Fusi 1994; see also Leibold and Kempter 2006; Barrett and van Rossum 2008; Huang and Amit 2010). Memory lifetimes may be extended by considering either sparse coding at the population level (Tsodyks and Feigel’man 1988) or complex models of synaptic plasticity in which synapses can express metaplasticity (changes in internal states) without necessarily expressing plasticity (changes in strength) (Fusi et al. 2005; Leibold and Kempter 2008; Elliott and Lagogiannis 2012; Lahiri and Ganguli 2013). Two previous studies have examined complex models of synaptic plasticity operating in concert with sparse coding (Leibold and Kempter 2008; Rubin and Fusi 2007). For a discussion of the possible roles of the persistence and transience of memories and the synaptic mechanisms underlying synaptic stability, see, for example, Richards and Frankland (2017), and Rao-Ruiz et al. (2021).

We have proposed integrate-and-express models of synaptic plasticity in which synapses act as low-pass filters in order to control fluctuations in developmental patterns of synaptic connectivity (Elliott 2008; Elliott and Lagogiannis 2009). We have also applied these complex models of synaptic plasticity to memory formation, retention and longevity with discrete synapses (Elliott and Lagogiannis 2012), finding that they outperform cascade models (Fusi et al. 2005) in most biologically relevant regions of parameter space (Elliott 2016b). In this paper, we consider the role of sparse coding in the memory dynamics of a filter-based model. For comparison, we also consider the cascade model (Fusi et al. 2005), the serial synapse model (Leibold and Kempter 2008; Rubin and Fusi 2007) and a model of simple synapses (Tsodyks 1990) using our protocols.

Our paper is organised as follows. In Sect. 2, we present our general approach by describing the two memory storage protocols that we study, considering two different definitions of memory lifetimes, and obtaining general, model-independent results. Then, in Sect. 3, we consider both simple and complex models of synaptic plasticity, obtaining the analytical results required to study memory lifetimes in detail. We compare and contrast results for memory lifetimes in simple and complex models in Sect. 4. Finally, in Sect. 5, we briefly discuss our results.

## General approach and formulation

We provide a convenient list of the most commonly used mathematical symbols and their meanings, excluding those that appear in the appendices, in Table 1.

**Table 1:** List of frequently used mathematical symbols and their meanings (excluding those in Appendix B)

Symbol	Meaning
*P*	Number of neurons in memory system
*N*	Number of synaptic inputs received by each neuron
$\mathscr {N}=NP$	Total number of synapses in memory system
*r*	Rate of Poisson process for memory storage
$S_i(t)$	Strength of synapse *i*, $i=1,\ldots ,N$, at time $t \ge 0$ s for a typical perceptron in the population
$\underline{\xi }^\alpha$	Memory $\alpha$, $\alpha = 0,1,2,\ldots$, to be stored by a single perceptron at Poisson storage step $\alpha$
$h(t) = h_{\underline{\xi }^0}(t)$	Memory signal for the tracked memory stored at $t=0$ s
$h_\alpha$	Memory signal for the tracked memory immediately after the storage of memory $\alpha$
$h_0$	$h_0 = h(0)$, the initial perceptron activation induced by the tracked memory $\underline{\xi }^0$
$\mu (t)$, $\sigma (t)^2$, $\mathrm {SNR}(t)$	Mean, variance and signal-to-noise ratio of *h*(*t*), the single-perceptron memory signal
$\tau _{\mathrm {snr}}$	SNR memory lifetime of a typical memory, for a single perceptron
$\tau _{\mathrm {mfpt}}(h_0)$	MFPT memory lifetime conditioned on a definite activation $h_0$ induced by a definite, tracked memory
$\vartheta$	Perceptron’s firing threshold
$\tau _{\mathrm {mfpt}}$	Conditional MFPT $\tau _{\mathrm {mfpt}}(h_0)$ averaged over all tracked memories with $h_0 > \vartheta$
$\sigma _{\mathrm {fpt}}^2$	Variance in the FPT
*A*(*h*), *B*(*h*)	Jump moments in Fokker–Planck equation
$\zeta$	Level of spontaneous electrical activity, with $0 \le \zeta < 1$
*f*, *g*	Probabilities of evoked (rather than spontaneous) pre- and postsynaptic (respectively) activities; $f \equiv g$
*s*	Number of internal synaptic states for each of the two possible states of synaptic strength
$\mathbb {M}_+$, $\mathbb {M}_-$; $\mathbb {M}$	Potentiating and depressing matrices describing transitions in a single synapse’s state; $\mathbb {M} = {\textstyle \frac{1}{2}}\left( \mathbb {M}_+ + \mathbb {M}_- \right)$
$\mathbb {K}_\pm$; $\mathbb {K}$	$\mathbb {K}_\pm = (1-f) \mathbb {I} + f \mathbb {M}_\pm$, $\mathbb {I}$ being the identity matrix; $\mathbb {K} = {\textstyle \frac{1}{2}}\left( \mathbb {K}_+ + \mathbb {K}_- \right)$
$\mathbb {T}_n$	Operator describing simultaneous changes in *n* synapses’ states at each (average) non-tracked memory storage step
$\underline{A}_n$	Normalised unit eigenstate of $\mathbb {T}_n$, giving the joint equilibrium probability distribution of *n* synapses’ states
$\underline{\Omega }$	$\underline{\Omega }^{\mathrm {T}} = \left( {-} \underline{1}^\mathrm {T} \, | \, {+} \underline{1}^{\mathrm {T}} \right)$, the vector by which to weight a synapse’s internal states by their strengths
$P_{\mathrm {eff}}$	Number of neurons in the population of *P* neurons that experience evoked activity during tracked memory storage
$\mu _p(t)$, $\sigma _p(t)^2$, $\mathrm {SNR}_p(t)$	Mean, variance and signal-to-noise ratio of the population memory signal
$\tau _{\mathrm {pop}}$	SNR memory lifetime of a typical memory for the population of neurons
*p*, $\psi$	Probability *p* of synaptic updates for a simple stochastic updater (SU) synapse; $\psi = f p$ is a convenient shorthand
$\kappa _2$	$\kappa _2 = \psi / (2 - \psi )$, the equilibrium correlation between pairs of SU synapses’ strengths in the Hebb protocol
$B_0$	$B_0 = B(0)/(\psi g)$, so that part of *B*(*h*) that is independent of *h*, up to scaling factors
$N_{\mathrm {eff}}$	Number of a single perceptron’s synapses that experience evoked presynaptic activity during tracked memory storage
$\Theta$	Synaptic filter threshold
$X^{\mathrm {opt}}$	For any parameter *X*, the label “opt” indicates that value of *X* that maximises $\tau _{\mathrm {snr}}$ or $\tau _{\mathrm {pop}}$
$\tau _{\mathrm {snr}}^{\mathrm {opt}}$, $\tau _{\mathrm {pop}}^{\mathrm {opt}}$	Maximum values of $\tau _{\mathrm {snr}}^{\mathrm {opt}}$ or $\tau _{\mathrm {pop}}^{\mathrm {opt}}$, optimised with respect to some parameter *X*

### Memories and memory lifetimes

We consider a population of *P* neurons forming a memory system, perhaps performing association or auto-association tasks. Let each neuron receive *N* synaptic connections from *N* other neurons that are randomly selected from the entire population. Fully recurrent connectivity would imply that $N = P - 1$ (excluding self-connections) but in general $N \ll P$. Other than the requirement that $N < P$, *N* may be regarded as mathematically independent of *P*. Memories are stored sequentially, one after the other, by this memory system. We take them to be stored at times $t \ge 0$ s governed by a Poisson process of rate *r* Hz. This continuous time approach is more realistic than a discrete time approach in which memories are stored at uniformly spaced time steps. Due to ongoing synaptic plasticity driven by the storage of later memories, the synaptic patterns that embody earlier memories may be degraded, so that the fidelity of recall of earlier memories may fall over time, ultimately falling to an equilibrium or background level of complete amnesia. It is typical in these scenarios to track the fidelity of recall of the first memory as subsequent memories are stored. This first memory is taken to be stored at time $t=0$ s on the background equilibrium probability distribution of synaptic strengths.

In previous work, we have focused on a single neuron, or perceptron, in such a system and have examined its recall of stored memories. Here, in a sparse population coding context, we must consider the collective dynamics of the entire population of neurons, but these collective dynamics are nevertheless driven by synaptic processes occurring at the level of single perceptrons in the system. Considering, then, a single perceptron in this population, let its *N* synapses have strengths $S_i(t) \in \{-1, +1\}$, $i=1, \ldots , N$, at time $t \ge 0$ s. These two strength states should be thought of as low and high rather than inhibitory and excitatory. As memories are presented to the system for storage, the perceptron is exposed to synaptic inputs characterised by the *N*-dimensional vectors $\underline{\xi }^\alpha$, $\alpha = 0, 1, 2, \ldots$, where $\alpha$ indexes the memories. The component $\xi _i^\alpha$ represents the input through synapse *i* during the presentation of memory $\alpha$, and for simplicity we assume that these components are independent between synapses and across memories.

In response to each of these memory vectors, the perceptron must generate the correct activation or output. With inputs $x_i$ through its *N* synapses, the perceptron’s activation is defined as usual by

1 $$\begin{aligned} h_{\underline{x}}(t) = \frac{1}{N} \sum _{i=1}^N x_i \, S_i(t). \end{aligned}$$

The perceptron’s output is some possibly nonlinear function of its activation, where this output can correspond to spontaneous activity under conditions of no (or spontaneous) input. We track the fidelity of recall of the first memory $\underline{\xi }^0$ by examining the perceptron’s activation upon re-presentation (but not re-storage) of this memory at later times $t > 0$ s. We refer to $h(t) \equiv h_{\underline{\xi }^0}(t)$ as the tracked memory signal or just the memory signal. The dynamics of *h*(*t*) will determine the lifetime of memory $\underline{\xi }^0$, at least as far as this single perceptron’s capacity to generate the correct output upon re-presentation of $\underline{\xi }^0$ is concerned. Of course, we are not interested in the lifetime of any particular tracked memory $\underline{\xi }^0$ stored on any particular pattern of synaptic connectivity and subject to any particular sequence of subsequent, non-tracked memories $\underline{\xi }^\alpha$, $\alpha > 0$, stored at any particular set of Poisson-distributed times $0< t_1< t_2< t_3 < \cdots$. Rather, we are interested only in the lifetime of a typical tracked memory subject to a typical sequence of later memories. Thus, we consider only the statistical properties of *h*(*t*) when suitably averaged over all memories.

Memory lifetimes may be defined in a variety of ways using these statistical properties. The simplest definition is to consider the mean and variance of *h*(*t*)

2a $$\begin{aligned} \mu (t)&= \mathsf {E}[ h(t) ], \end{aligned}$$

2b $$\begin{aligned} \sigma (t)^2&= \mathsf {Var}[ h(t) ], \end{aligned}$$

define the signal-to-noise ratio (SNR) as:

3 $$\begin{aligned} \mathrm {SNR}(t) = \left[ \mu (t) - \mu (\infty ) \right] /\sigma (t), \end{aligned}$$

and then define the memory lifetime as that value of *t*, call it $\tau _{\mathrm {snr}}$, that is the (largest) finite, non-negative solution of the equation $\mathrm {SNR}(\tau _{\mathrm {snr}}) = 1$ when this solution exists; otherwise, we set $\tau _{\mathrm {snr}} = 0$ s (Tsodyks 1990). This is the last time at which $\mu (t)$ is distinguishable from its equilibrium value $\mu (\infty )$ at the level of one standard deviation. Although an “ideal observer” approach to defining memory lifetimes has also been considered (Fusi et al. 2005; Lahiri and Ganguli 2013), it is essentially equivalent to the SNR approach (Elliott 2016b).

The activation *h*(*t*) provides a direct read-out of the perceptron’s response to the re-presentation of $\underline{\xi }^0$ at later times, and would correspond to a neuron’s membrane potential in a more realistic, integrate-and-fire model. By focusing on this read-out of the perceptron’s state, we are naturally led to consider the first passage time (FPT) for the perceptron’s activation to fall below firing threshold, and thus to consider the mean first passage time (MFPT) for this process, which is the mean taken over all tracked and non-tracked memories (Elliott 2014). We may then define an alternative memory lifetime, call it $\tau _{\mathrm {mfpt}}$, as this MFPT for the perceptron’s activation in response to re-presentation of a typical tracked memory to fall below firing threshold. We have extensively discussed and contrasted the SNR and FPT approaches to defining memory lifetimes elsewhere (Elliott 2014, 2016a, 2017a). In essence, SNR memory lifetimes are only valid in asymptotic, typically large *N* regimes, while FPT memory lifetimes are valid in all regimes. SNR lifetimes must therefore be interpreted with caution.

To compute FPT lifetimes, we require $\mathsf {Prob}[ h_{\alpha +1} | h_\alpha ]$, the transition probability describing the probability that the perceptron’s activation (in response to re-presentation of the tracked memory) is $h_{\alpha +1}$ immediately after the storage of average non-tracked memory $\underline{\xi }^{\alpha +1}$, given that its activation is $h_\alpha$ immediately before the memory’s storage. This transition probability is most easily computed in simple models of synaptic plasticity, for which it is independent of the memory storage step (Elliott 2014, 2017a, 2019). This independence arises because simple synapses with only two strength states are “stateless” (Elliott 2020), having no internal states and not enough strength states to carry information between consecutive memory storage steps. In this case, all the probabilities $\mathsf {Prob}[ h_{\alpha +1} | h_\alpha ]$ over all the possible, discrete values of $h_\alpha$ and $h_{\alpha +1}$ define the elements of a transition matrix in the perceptron’s activation between memory storage steps that is independent of the non-tracked memory storage step $\alpha + 1$ ($\alpha \ge 0$). We can then drop the index $\alpha$ and consider general elements $\mathsf {Prob}[ \, h \, | \, h' \, ]$ between any two possible values of the perceptron activation, *h* and $h'$. We will therefore examine FPT lifetimes only for simple synapses, and SNR memory lifetimes for both simple and complex synapses, but with the understanding that SNR results must be interpreted cautiously. With the transition probabilities $\mathsf {Prob}[ \, h \, | \, h' \, ]$ being independent of the memory storage step, and with the storage of the definite tracked memory $\underline{\xi }^0$ inducing the definite activation $h_0$ immediately after its storage, the FPT lifetime of the memory $\underline{\xi }^0$ is the solution of the equation

4 $$\begin{aligned} \tau _{\mathrm {mfpt}} ( h_0 ) = \frac{1}{r} + \sum _{h > \vartheta } \tau _{\mathrm {mfpt}} ( h ) \, \mathsf {Prob}[ \, h \, | \, h_0 \,], \end{aligned}$$

where transitions to activations below the firing threshold $\vartheta$ are disallowed (Elliott 2014; see van Kampen 1992, for a general discussion). Equation (4) generalises in the obvious way to an integral equation when *h* can be regarded as a continuous rather than discrete variable. Solving Eq. (4) for $\tau _{\mathrm {mfpt}}(h_0)$ for all values of $h_0 > \vartheta$ entails solving a linear system involving the perceptron activation transition matrix. We therefore refer to Eq. (4) for simplicity but somewhat inaccurately as a matrix equation, to distinguish it from its integral equation equivalent in the continuum limit. This matrix or integral equation (MIE) approach to FPTs is exact. We may also consider an approximation involving the Fokker–Planck equation (FPE) approach by computing the jump moments induced by $\mathsf {Prob}[ \, h \, | \, h' \, ]$. Because memories are stored as a Poisson process, the jump moments are simply

5a $$\begin{aligned} A(h')&= \mathsf {E}[ ( h - h' )^1 \, | \, h' \, ], \end{aligned}$$

5b $$\begin{aligned} B(h')&= \mathsf {E}[ ( h - h' )^2 \, | \, h' \, ], \end{aligned}$$

where the expectation values are calculated with respect to $\mathsf {Prob}[ \, h \, | \, h' \, ]$. Then, standard methods (Elliott 2014; see van Kampen 1992, in general) give the MFPT as the solution of the equation:

6 $$\begin{aligned} - \frac{1}{r} = A(h_0) \frac{{d}}{{d} h_0} \tau _{\mathrm {mfpt}}(h_0) + \frac{1}{2} B(h_0) \frac{{d}^2}{{d} h_0^2} \tau _{\mathrm {mfpt}}(h_0), \end{aligned}$$

subject to the boundary condition $r \tau _{\mathrm {mfpt}}(\vartheta ) = 0$. Equations similar to Eqs. (4) and (6) give the higher-order FPT moments (Elliott 2019). Given $\tau _{\mathrm {mfpt}}( h_0 )$, we obtain $\tau _{\mathrm {mfpt}}$ by averaging over the distribution of $h_0$ (for values of $h_0 > \vartheta$), corresponding to averaging over the tracked memory $\underline{\xi }^0$, i.e. $\tau _{\mathrm {mfpt}} = \langle \tau _{\mathrm {mfpt}}( h_0 ) \rangle _{h_0 > \vartheta }$.

### Hebb protocol

We adopt and adapt the memory storage protocol employed by Leibold and Kempter (2008). Their memory system performs an association task. Within the population of *P* neurons, a sub-population of “cue” neurons is required to activate a sub-population of “target” neurons. Synapses from cue to target neurons experience potentiating induction signals during memory storage, while those from target to cue experience depressing induction signals; all other synapses do not experience plasticity induction signals. Although Leibold and Kempter (2008) do allow for the possibility of some overlap between cue and target sub-populations, this will not be relevant here. The storage of different memories involves different cue and target sub-populations, so that the entire population of *P* neurons will be involved in storing many memories over time. If cue and target sub-populations are of equal size, as we assume, then potentiation and depression processes are equally balanced on average. This assumption stands in lieu of realistic neuron models, in which we expect (Elliott 2016a) synaptic plasticity to be dynamically regulated to move to stable dynamical fixed points in which such balancing is achieved automatically (Bienenstock et al. 1982; Burkitt et al. 2004; Appleby and Elliott 2006).

While Leibold and Kempter (2008) consider activities $\xi _i^\alpha \in \{ 0, 1 \}$, corresponding to inactive ($\xi _i^\alpha = 0$) and active ($\xi _i^\alpha = 1$) input neurons, we will consider the more general case of $\xi _i^\alpha \in \{ \zeta , 1 \}$, with $0 \le \zeta < 1$, where $\xi _i^\alpha = \zeta$ represents a spontaneous, non-evoked, or background level of activity for an input neuron that is in neither cue nor target sub-populations, while $\xi _i^\alpha = 1$ represents evoked activity from a cue or target input. We often refer below to active and inactive inputs or neurons, with the understanding that we mean evoked activity and spontaneous activity, respectively. Because synaptic plasticity occurs only between cue and target neurons, synapses between a pre- or postsynaptic neuron that is only spontaneously active do not undergo synaptic plasticity. This accords with our expectations from known physiology: protocols for long-term potentiation (LTP; Bliss and Lømo 1973) and long-term depression (LTD; Lynch et al. 1977) require sustained bouts of evoked electrical activity rather than just spontaneous levels of activity. On a broadly BCM view of synaptic plasticity (Bienenstock et al. 1982), we would expect two thresholds for synaptic plasticity: as activity levels ramp up from spontaneous to weak to strong tetanisation, plasticity switches from none to LTD to LTP. Since synaptic plasticity can only occur between pairs of active, synaptically coupled neurons in this scenario, we refer to it as the Hebb protocol: Hebbian synaptic plasticity is typically understood to mean activity-dependent, bidirectional synaptic plasticity between active pre- and postsynaptic neurons. Although spontaneous activity has by assumption no impact on synaptic plasticity here, it nevertheless has a direct impact on *h*(*t*).

For a particular perceptron, let the probability that it is active during the storage of any particular memory be *g*. Since the perceptron could be part of either cue or target sub-populations, the probability that it is either cue or target during the storage of a memory is just ${\textstyle \frac{1}{2}}g$. The probability that any one of its synaptic inputs is active during memory storage is also just *g*. However, for the purposes of clarity it is convenient to distinguish between these two probabilities, so we denote the probability that an input is active as *f* ($\equiv g$). In this way, the appearance of a factor of *g* indicates a global, postsynaptic factor due to the perceptron, or postsynaptic cell, being in the cue or target population, while a factor of *f* indicates a local, presynaptic factor due to an input being in the cue or target population. The probability *g*, or *f*, controls the sparseness of the memory representation in this memory system. Considering just a single perceptron, if it is neither cue nor target, then none of its synapses can experience plasticity induction signals. If it is a cue, then only those inputs that correspond to target cells (if any) experience plasticity induction signals, and specifically depressing signals. If it is a target, then similarly only cue inputs experience induction signals, and so only potentiating signals. Without loss of generality, we may therefore just assume that during memory storage, an active perceptron’s active inputs are either all cue or all target neurons. This simplifying assumption effectively doubles on average the rate of plasticity induction signals experienced by synapses compared to the scenario in which the perceptron’s active inputs could represent a combination of cue and target neurons. We could therefore just scale *f* accordingly.

**Fig. 1:**
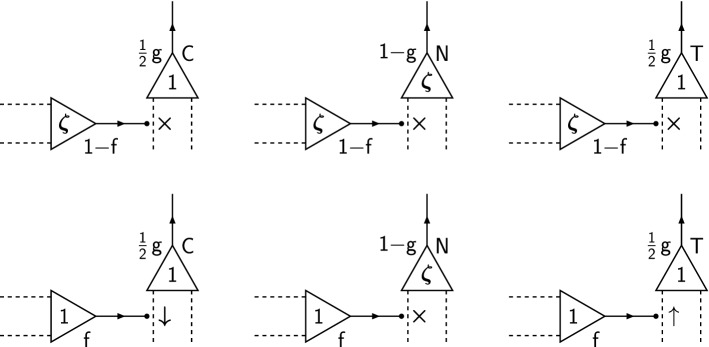
Schematic illustration of the Hebb protocol for memory storage. Six pairs of synaptically coupled neurons are shown. Each cell body is represented by a triangle, with the value ($\zeta$ or 1) inside the triangle indicating the neuron’s activity during memory storage. A neuron’s axon is denoted by a directed line, while two of its dendrites are denoted by the dashed lines. Synaptic coupling is indicated by a small black blob where an axon terminates on a dendrite, with the symbol to the right of the blob indicating the direction of induced synaptic plasticity during memory storage (“$\uparrow$” indicates potentiation, “$\downarrow$” depression, and “$\times$” no change). The labels “C”, “N” or “T” attached to a postsynaptic cell body indicate that the neuron is a cue cell, neither a cue cell nor a target cell, or a target cell, respectively, in the population. Probabilities of presynaptic activity (*f* or $1-f$) are indicated, as are the joint probabilities of postsynaptic activity and specific role ($\frac{1}{2}g$ or $1-g$). The fact that an active presynaptic neuron synapsing on a cue or target cell always experiences the induction of depression or potentiation, respectively, reflects the simplifying assumption discussed in the main text

We summarise the Hebb protocol in Fig. 1, which schematically illustrates a sample of the population of pairs of pre- and postsynaptic neurons, showing all possible combinations of presynaptic activities and postsynaptic roles with their respective probabilities, together with the direction of synaptic plasticity induced by them.

To assess memory lifetimes under this protocol, we may track the ability of the cue sub-population to successfully evoke activity in the target sub-population. Considering a single perceptron in the target sub-population, we may obtain general expressions for $\mu (t)$ and $\sigma (t)^2$ in Eq. (2), where these expressions are independent of any particular model of synaptic plasticity. Because $h(t) = \frac{1}{N} \sum _{i=1}^N \xi _i^0 S_i(t)$ and similarly $h(t)^2 = \frac{1}{N^2} \sum _{i,j=1}^N \xi _i^0 \xi _j^0 S_i(t) S_j(t)$, their expectation values lead to

7a $$\begin{aligned} \mu (t)&= f \, \mathsf {E}\left[ S_1(t) \, | \, + \, \right] + (1 - f) \, \zeta \, \overbrace{ \mathsf {E}\left[ S_1(t) \, | \, {\times } \, \right] }^{\equiv 0}, \end{aligned}$$

7b $$\begin{aligned} \sigma (t)^2&= \frac{f + (1-f) \zeta ^2 - \mu (t)^2}{N} \nonumber \\&\quad + \frac{N-1}{N} \Big \{ f^2 \, \mathsf {E}\left[ S_1(t) S_2(t) \, | \, {+} {+} \, \right] \nonumber \\&\quad + 2 f (1-f) \, \zeta \, \mathsf {E}\left[ S_1(t) S_2(t) \, | \, {+} {\times } \, \right] \nonumber \\&\quad + (1-f)^2 \zeta ^2 \, \mathsf {E}\left[ S_1(t) S_2(t) \, | \, {\times } {\times } \, \right] - \mu (t)^2 \Big \}, \end{aligned}$$

where we could pick any synapse *i* in Eq. (7a) and any distinct pair of synapses *i* and *j* in Eq. (7b) but we restrict without loss of generality to $i=1$ and $j=2$. In these equations, we condition on whether a synapse has experienced a potentiating induction signal (“$+$”) with probability *f* or not (“$\times$”) with probability $1-f$, during the storage of $\underline{\xi }^0$. For the models of synaptic plasticity that we consider below, the (marginal) equilibrium probability distribution of any single synapse’s strength is uniform, or $\mathsf {Prob}[ S_i (\infty ) = \pm 1 ] = {\textstyle \frac{1}{2}}$, so that if a synapse does not experience a plasticity induction signal during the storage of $\underline{\xi }^0$, then $\mathsf {E}\left[ S_i(t) \, | \, {\times } \, \right] \equiv 0$ at $t=0$ s and this remains true for all times $t \ge 0$ s when potentiation and depression processes are treated symmetrically, as indicated in Eq. (7a). However, for the pairwise correlations in Eq. (7b) that condition on one or both synapses not having experienced an induction signal, the expectation values do not vanish under the Hebb protocol. This is because of the higher-order equilibrium correlational structure induced by the fact that it is impossible for some of the synapses of an active neuron to experience potentiating induction signals while others experience depressing induction signals during the storage of the same memory under the Hebb protocol.

We may obtain general expressions for the expectation values in Eq. (7) by writing down the transition processes that govern changes in a single synapse’s strength or simultaneous changes in a pair of synapses’ strengths. Let each synapse have *s* possible internal states for each of its two possible strengths $\pm 1$, so that the possible state of a synapse is described by a 2*s*-dimensional vector, with the internal states for strength $-1$ (respectively, $+1$) corresponding to the first (respectively, last) *s* components. Given the stochastic nature of the plasticity induction signals, this vector defines a joint probability distribution for a synapse’s combined strength and internal state. Let the transition matrix $\mathbb {M}_+$ implement the definite change in a synapse’s state in response to a potentiating induction signal, and $\mathbb {M}_-$ that for a depressing induction signal. We then determine the transition matrix governing the change in a single synapse’s state in response to the storage of a typical non-tracked memory by conditioning on all possible combinations of presynaptic activity and postsynaptic role. Defining $\mathbb {K}_\pm = (1 - f) \mathbb {I} + f \mathbb {M}_\pm$, where $\mathbb {I}$ is the identity matrix, this transition matrix is

8a $$\begin{aligned} \mathbb {T}_1 = (1 - g) \, \mathbb {I} + {\textstyle \frac{1}{2}}\, g \, \mathbb {K}_+ + {\textstyle \frac{1}{2}}\, g \, \mathbb {K}_-, \end{aligned}$$

or just $\mathbb {T}_1 = (1 - g) \, \mathbb {I} + g \, \mathbb {K}$, where $\mathbb {K} = (1 - f) \mathbb {I} + f \mathbb {M}$ with $\mathbb {M} = {\textstyle \frac{1}{2}}( \mathbb {M}_+ + \mathbb {M}_- )$. The three terms in Eq. (8a) arise from conditioning on the three possible perceptron roles in memory storage (determined by the global factor *g*), while the two terms in each of $\mathbb {K}_\pm$ arise from conditioning on the two possible levels of presynaptic activity (determined by the local factor *f*). Similarly, the transition operator that governs simultaneous changes in pairs of synapses’ states during typical non-tracked memory storage is

8b $$\begin{aligned} \mathbb {T}_2 = (1 - g) \, \mathbb {I} \otimes \mathbb {I} + {\textstyle \frac{1}{2}}\, g \, \mathbb {K}_+ \otimes \mathbb {K}_+ + {\textstyle \frac{1}{2}}\, g \, \mathbb {K}_- \otimes \mathbb {K}_-, \end{aligned}$$

with the generalisation to $\mathbb {T}_n$ for any number of synapses *n* being clear. The (marginal) equilibrium probability distribution of a single synapse’s state, denoted by $\underline{A}_1$, is the (normalised) eigenvector of $\mathbb {T}_1$ with unit eigenvalue, which is also just the unit eigenvector of $\mathbb {M}$. That for any pair of synapses, $\underline{A}_2$, corresponds to the unit eigenvector of $\mathbb {T}_2$. However, because $\mathbb {T}_2 \ne (1-g) \mathbb {I} \otimes \mathbb {I} + g \, \mathbb {K} \otimes \mathbb {K}$, then $\underline{A}_2 \ne \underline{A}_1 \otimes \underline{A}_1$. Rather, $\underline{A}_2$ must be explicitly computed as the unit eigenstate of ${\textstyle \frac{1}{2}}( \mathbb {K}_+ \otimes \mathbb {K}_+ + \mathbb {K}_- \otimes \mathbb {K}_- )$. It is this failure of factorisation that induces the non-trivial pairwise correlational structure in the equilibrium state.

Using $\mathbb {T}_1$ and $\mathbb {T}_2$, we may write down the conditional expectation values in Eq. (7). We define the vector $\underline{\Omega }^{\mathrm {T}} = \left( {-} \underline{1}^\mathrm {T} \, | \, {+} \underline{1}^{\mathrm {T}} \right)$, where $\mathrm {T}$ denotes the transpose and the *s*-dimensional vector $\underline{1}$ is a vector all of whose components are unity. This vector weights synaptic states according to their two possible strengths. Then,

9a $$\begin{aligned} \mathsf {E}\left[ S_1(t) \, | \, + \, \right] = \underline{\Omega }^{\mathrm {T}} e^{\left( \mathbb {T}_1 - \mathbb {I} \right) r t} \, \mathbb {M}_+ \underline{A}_1, \end{aligned}$$

and

9b $$\begin{aligned}&\mathsf {E}\left[ S_1(t) S_2(t) \, | \, {+} {+} \, \right] \nonumber \\&\quad = \big ( \underline{\Omega }^{\mathrm {T}} \otimes \underline{\Omega }^{\mathrm {T}} \big ) e^{\left( \mathbb {T}_2 - \mathbb {I} \otimes \mathbb {I} \right) r t} \left( \mathbb {M}_+ \otimes \mathbb {M}_+ \right) \underline{A}_2, \end{aligned}$$

and for the other two pairwise expectation values in Eq. (7b), we replace $\mathbb {M}_+ \otimes \mathbb {M}_+$ in Eq. (9b) by $\mathbb {M}_+ \otimes \mathbb {I}$ for ${+} {\times }$ and $\mathbb {I} \otimes \mathbb {I}$ for ${\times } {\times }$. Since $\mathbb {T}_1 - \mathbb {I} = f g \left( \mathbb {M} - \mathbb {I} \right)$, we have $\mu (t) = f \, \underline{\Omega }^{\mathrm {T}} e^{\left( \mathbb {M} - \mathbb {I} \right) f g r t} \, \mathbb {M}_+ \underline{A}_1$, so that sparse coding just introduces a multiplicative factor of *f* and scales the rate *r* by the product *fg* in $\mu (t)$. In the equilibrium limit, by definition $\exp [(\mathbb {T}_n - \mathbb {I} \otimes \cdots \otimes \mathbb {I})rt] \, \underline{v} \rightarrow \underline{A}_n$ for any state $\underline{v}$ corresponding to a probability distribution, as $t \rightarrow \infty$. Hence, $\mu (\infty ) = f \, \underline{\Omega }^{\mathrm {T}} \underline{A}_1 \equiv 0$, which always follows when potentiation and depression processes are treated symmetrically. For the equilibrium variance, we obtain

10 $$\begin{aligned} \sigma (\infty )^2&= \frac{f + (1-f) \zeta ^2}{N} \nonumber \\&\quad + \frac{N-1}{N} \left[ f + (1 - f) \zeta \right] ^2 \big ( \underline{\Omega }^{\mathrm {T}} \otimes \underline{\Omega }^{\mathrm {T}} \big ) \underline{A}_2. \end{aligned}$$

The second, covariance term does not in general vanish because of the equilibrium synaptic pairwise correlations.

These general results allow us to obtain SNR lifetimes when the matrices $\mathbb {M}_\pm$ are specified for any particular model of synaptic plasticity. We defer the derivation of the transition matrix elements $\mathsf {Prob}[ \, h \, | \, h' \, ]$ that are required for FPT lifetimes until we explicitly discuss simple models of synaptic plasticity in Sect. 3.1.

### Hopfield protocol

Although the Hebb protocol is intuitive as a means of exploring memory lifetimes in an associative memory system, its non-trivial equilibrium distribution of synaptic states is awkward. To avoid this awkwardness, we may consider an alternative protocol that is nevertheless equivalent to the Hebb protocol in the limit of small *fgN*. We first define the protocol and then demonstrate the equivalence.

During memory storage, instead of defining cue and target sub-populations, we now specify the entire activity pattern, representing a memory, across the whole population of neurons. We allow these activities to take values from the set $\{ -1, -\zeta , +\zeta , +1 \}$ with probabilities $\{ {\textstyle \frac{1}{2}}f, {\textstyle \frac{1}{2}}(1-f), {\textstyle \frac{1}{2}}(1-f), {\textstyle \frac{1}{2}}f \}$. Here, the values $\pm 1$ represent evoked activity (the neuron is involved in memory storage), with $+1$ (respectively, $-1$) representing a strongly (respectively, weakly) tetanising stimulus in the usual LTP (respectively, LTD) sense. In contrast, the values $\pm \zeta$ represent spontaneous activity (the neuron is not involved in memory storage). For a single perceptron, this amounts to specifying memory vectors $\underline{\xi }^\alpha$ with components $\xi _i^\alpha$ taking one of these four values, and also specifying the perceptron’s required output in response to an input vector, where this output is drawn from the same set with the same probabilities, but with *f* replaced by *g* as usual. We can track the perceptron’s activation when its required output is either $+1$ or $-1$, but by symmetry its activation would differ only by a sign between these two cases, so for concreteness we just take the required output to be $+1$ during the storage of $\underline{\xi }^0$. As with the Hebb protocol, a synapse does not experience a plasticity induction signal if its presynaptic input or the postsynaptic perceptron itself is only spontaneously active. However, if both input and perceptron are active, then the synapse experiences a plasticity induction signal, either potentiating if both activities are the same or depressing if different. This is just the standard Hopfield rule (Hopfield 1982), so we refer to this protocol as the Hopfield protocol: we obtain a pattern of synaptic plasticity induction signals in response to evoked activity that is identical to the standard Hopfield rule, but supplemented by the presence of spontaneous activity that does not induce synaptic plasticity.

**Fig. 2:**
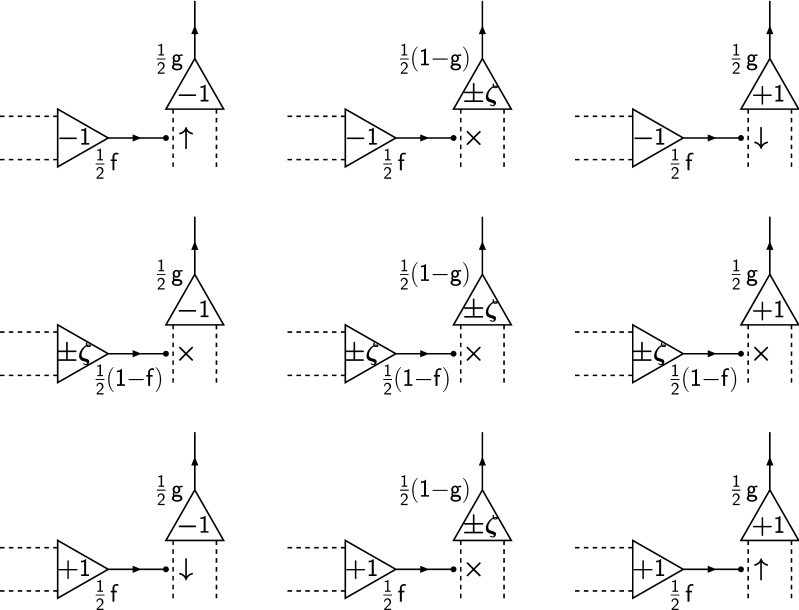
Schematic illustration of the Hopfield protocol for memory storage. The format of this figure is essentially identical to that for the Hebb protocol in Fig. 1, except that labels indicating postsynaptic roles are not required. To avoid duplication, spontaneously active neurons are shown with both possible spontaneous activity levels, $\pm \zeta$; the corresponding probability is for each of these levels rather than for both

Figure 2 summarises the Hopfield protocol, showing all allowed combinations of pre- and postsynaptic activities during memory storage, together with their associated probabilities and induced plasticity induction signals. Depressing and potentiating induction signals both occur with the same overall probability ${\textstyle \frac{1}{2}}f g$ as in the Hebb protocol.

Computing $\mu (t)$ and $\sigma (t)^2$ for the Hopfield protocol and using the various symmetries $\mathsf {E}\left[ S_1(t) \, | \, + \, \right] = - \mathsf {E}\left[ S_1(t) \, | \, - \, \right]$, $\mathsf {E}\left[ S_1(t) S_2(t) \, | \, {+} {+} \, \right] = \mathsf {E}\left[ S_1(t) S_2(t) \, | \, {-} {-} \, \right]$, etc., we obtain

11a $$\begin{aligned} \mu (t)&= f \, \mathsf {E}\left[ S_1(t) \, | \, + \, \right] , \end{aligned}$$

11b $$\begin{aligned} \sigma (t)^2&= \frac{f + (1-f) \zeta ^2 - \mu (t)^2}{N} \nonumber \\&\quad + \frac{N-1}{N} \Big \{ f^2 \, \mathsf {E}\left[ S_1(t) S_2(t) \, | \, {+} {+} \, \right] \nonumber \\&\quad + (1-f)^2 \zeta ^2 \, \underbrace{\mathsf {E}\left[ S_1(t) S_2(t) \, | \, {\times } {\times } \, \right] }_{\equiv 0} - \mu (t)^2 \Big \}. \end{aligned}$$

These are structurally identical to the expressions in Eq. (7) for the Hebb protocol, except that the linear terms in $\zeta$ are absent because of cancellation. Had we instead used a single level $\zeta$ of spontaneous activity rather than the two levels $\pm \zeta$, we would have obtained identical linear terms, too. Writing down the transition operators $\mathbb {T}_1$ and $\mathbb {T}_2$ in the Hopfield protocol, we obtain

12a $$\begin{aligned} \mathbb {T}_1&= (1 - g) \, \mathbb {I} + g \, \mathbb {K}, \end{aligned}$$

12b $$\begin{aligned} \mathbb {T}_2&= (1 - g) \, \mathbb {I} \otimes \mathbb {I} + g \, \mathbb {K} \otimes \mathbb {K}, \end{aligned}$$

with immediate generalisation to $\mathbb {T}_n$. The (marginal) equilibrium distribution of a pair of synapses’ states is therefore determined by the unit eigenstate of $\mathbb {K} \otimes \mathbb {K}$ and thus of $\mathbb {M} \otimes \mathbb {M}$, and so is just $\underline{A}_2 = \underline{A}_1 \otimes \underline{A}_1$; again, generalisation to $\underline{A}_n$ is immediate. The result is that all conditional expectation values involving at least one synapse that does not experience a plasticity induction event during the storage of $\underline{\xi }^0$ vanish, when potentiation and depression processes are treated symmetrically. So, whether we use four-level or three-level activities in the Hopfield protocol, the $\zeta$-dependent contributions to the covariance term in Eq. (11b) drop out, as indicated, so that the variance is affected only by the $\zeta ^2$ term in the first term on the right-hand side (RHS) of Eq. (11b). Moreover, the covariance term vanishes entirely in the large *t*, equilibrium limit, since $\mathsf {E}\left[ S_1(t) S_2(t) \, | \, {+} {+} \, \right] \rightarrow \big ( \underline{\Omega }^{\mathrm {T}} \underline{A}_1 \big )^2 \equiv 0$, so that $\sigma (\infty )^2 = [ f + (1-f) \zeta ^2 ] / N$.

The equivalence of the Hebb and Hopfield protocols in the limit of small *fgN* is now clear. The corresponding transition matrices $\mathbb {T}_1$ are in any case identical for both protocols, and hence so are the means. For $\mathbb {T}_2$, in both protocols we have that

13 $$\begin{aligned} \mathbb {T}_2 - \mathbb {I} \otimes \mathbb {I} = f g \left[ \left( \mathbb {M} - \mathbb {I} \right) \otimes \mathbb {I} + \mathbb {I} \otimes \left( \mathbb {M} - \mathbb {I} \right) \right] + \mathscr {O}(f^2 g),\nonumber \\ \end{aligned}$$

and for general $\mathbb {T}_N$ the $\mathscr {O}(f g)$ term on the RHS contains *N* terms, each of which contains $N-1$ factors of $\mathbb {I}$ and just one factor of $\mathbb {M} - \mathbb {I}$. This structure reflects the fact that in the limit of small *fgN*, at most one of the perceptron’s synapses experiences a plasticity induction signal, regardless of the protocol. The corresponding unit eigenstate of $\mathbb {T}_N$ in this limit is just $\underline{A}_1 \otimes \cdots \otimes \underline{A}_1$, regardless of the protocol. Therefore, in the small *fgN* limit, the equilibrium distribution of synaptic states in the Hebb protocol reduces to that in the Hopfield protocol, and all statistical properties of *h*(*t*) must therefore also reduce in the same way. The Hopfield protocol therefore offers a way of extrapolating the small *fgN* behaviour of the Hebb protocol to larger *f* without the awkwardness of the Hebb protocol’s equilibrium structure in this regime. Furthermore, the simpler form of the results in the Hopfield protocol allow us to use it to extract the scaling properties of memory lifetimes as a function of small *f* (or *g*) in both protocols.

For the non-sparse-coding case of $f=1$, spontaneous activity does not contribute to the Hopfield protocol’s dynamics, and we recover precisely the Hopfield model with discrete-state synapses. For $f<1$, we expand the possible activities of neurons to allow for spontaneously active neurons that are not involved in memory storage. Thus, although the Hopfield protocol provides a convenient tool for examining the small *fgN* limit of the Hebb protocol, we also regard the Hopfield protocol as a fully fledged protocol in its own right, because it constitutes a very natural way of examining sparse coding with a Hopfield plasticity rule.

### Population memory lifetimes

So far we have focused on the memory dynamics of a single perceptron. We now consider the memory dynamics of the entire population of *P* neurons. We do this only for the Hopfield protocol for simplicity. The tracked memory will evoke activity in a sub-population of on average *gP* neurons. In an experimental protocol, during the storage of the tracked memory we can at least in principle explicitly identify all those neurons that are active, and then subsequently track all their activities during later re-presentations of the tracked memory. Because of synaptic coupling between these tracked neurons and the other on average $(1-g)P$ neurons, spontaneous activity in the other neurons will affect and potentially degrade the activation of the tracked neurons upon re-presentation of the tracked memory, affecting the tracked neurons’ ability to read out the tracked memory. But, as we are only concerned with the tracked neurons’ read-out of the tracked memory, we do not need to explicitly track the activities of all these other neurons: their activities do not directly form part of the memory signal from the tracked neurons.

In the Hopfield protocol, a tracked neuron will by definition have an output of $+1$ or $-1$ during memory storage. For a single perceptron, we focused on an output of $+1$ without loss of generality. A perceptron with an initial output of $-1$ will have identical dynamics to one with an initial output of $+1$, except that the activation will be reversed in sign. Therefore, we can just define the memory signal for any active perceptron to be $\pm h(t)$, depending on this sign. Denoting the moment generating function (MGF) of $+h(t)$ for a tracked neuron with an initial output of $+1$ by $\mathscr {M}(z;t)$, the MGF for $-h(t)$ for a tracked neuron with an initial output of $-1$ will also be just $\mathscr {M}(z;t)$. All tracked neurons therefore have the same MGF for their memory signals.

Suppose that $P_{\mathrm {eff}}$ neurons form the sub-population that stores the tracked memory, where $P_{\mathrm {eff}}$ is binomially distributed with parameter *P* and probability *g*. Although these neurons’ activations will not in general evolve independently, as an extremely coarse approximation we assume that their activations do evolve independently during subsequent memory storage (cf. Rubin and Fusi 2007). Population memory lifetimes obtained from this simplifying assumption will therefore only be theoretical, and perhaps very loose, upper bounds on exact memory lifetimes. With this simplification, the MGF for the memory signal from the tracked sub-population is then $\left[ \mathscr {M}(z;t) \right] ^{P_{\mathrm {eff}}}$, by independence. Averaging over $P_{\mathrm {eff}}$, the MGF of the population memory signal is then just $\left[ (1-g) + g \mathscr {M}(z;t) \right] ^P$. The mean, $\mu _p(t)$, and variance, $\sigma _p(t)^2$, of this population signal follow directly. Ignoring covariance terms (or considering the limit $t \rightarrow \infty$ for the variance), we have $\mu _p(t) = g P \mu (t)$ and $\sigma _p(t)^2 \approx g P \sigma (t)^2$, where $\mu (t)$ and $\sigma (t)^2$ are the single-perceptron mean and variance above.^1^ Hence, the population SNR, $\mathrm {SNR}_p(t) = \left[ \mu _p(t) - \mu _p(\infty ) \right] / \sigma _p(t)$, is just scaled by the factor $\sqrt{g P}$ relative to the single-perceptron SNR, so

14 $$\begin{aligned} \mathrm {SNR}_p(t) \approx \sqrt{g P} \; \mathrm {SNR}(t). \end{aligned}$$

The population SNR memory lifetime, which we denote by $\tau _{\mathrm {pop}}$, is then the solution of $\mathrm {SNR}_p(\tau _{\mathrm {pop}}) = 1$. With $\sigma (t)^2 \approx [f + (1-f) \zeta ^2] / N$ in the Hopfield protocol, $\mathrm {SNR}_p(t)$ depends on $\mathscr {N} = N P$, the total number of synapses in the memory system, but it also contains the additional factor of $\sqrt{g}$ compared to $\mathrm {SNR}(t)$, which modifies scaling behaviour compared to single-perceptron results.

## Models of synaptic plasticity

### Simple synapses: the stochastic updater

The simplest model of synaptic plasticity to consider is one in which synapses lack any internal states so that $s=1$, and given a plasticity induction signal, they change strength (if possible) with some fixed probability *p* (Tsodyks 1990). Because a synapse just changes its strength stochastically in this model, we have called such a synapse a “stochastic updater” (SU; Elliott and Lagogiannis 2012). The underlying strength transition matrices are then

15 $$\begin{aligned} \mathbb {M}_+ = \begin{pmatrix} 1-p ,&{} \quad 0 \\ p ,&{}\quad 1 \end{pmatrix}, \, \, \mathbb {M}_- = \begin{pmatrix} 1 , &{}\quad p \\ 0 , &{}\quad 1-p \end{pmatrix}, \end{aligned}$$

and so

16 $$\begin{aligned} \mathbb {K}_+ = \begin{pmatrix} 1 - \psi , &{}\quad 0 \\ \psi , &{}\quad 1 \end{pmatrix}, \; \; \mathbb {K}_- = \begin{pmatrix} 1 , &{}\quad \psi \\ 0 , &{}\quad 1 - \psi \end{pmatrix}, \end{aligned}$$

where we define $\psi = f p$ for convenience.

The equilibrium distribution of a single synapse’s strength in both protocols is just the normalised unit eigenvector of $\mathbb {K}$, or $\underline{A}_1 = \frac{1}{2}\left( 1, 1 \right) ^{\mathrm {T}}$. For the Hopfield protocol, any pair of synapses’ strengths has the equilibrium distribution $\underline{A}_2 = \underline{A}_1 \otimes \underline{A}_1$. For the Hebb protocol, we require the unit eigenstate of ${\textstyle \frac{1}{2}}( \mathbb {K}_+ \otimes \mathbb {K}_+ + \mathbb {K}_- \otimes \mathbb {K}_- )$, which gives

17 $$\begin{aligned} \underline{A}_2&= \frac{1 \, {+} \, \kappa _2}{4} \left[ \begin{pmatrix} 1 \\ 0 \end{pmatrix} {\otimes } \begin{pmatrix} 1 \\ 0 \end{pmatrix} {+} \begin{pmatrix} 0 \\ 1 \end{pmatrix} {\otimes } \begin{pmatrix} 0 \\ 1 \end{pmatrix} \right] \nonumber \\&\quad + \frac{1 \, {-} \, \kappa _2}{4} \left[ \begin{pmatrix} 1 \\ 0 \end{pmatrix} {\otimes } \begin{pmatrix} 0 \\ 1 \end{pmatrix} {+} \begin{pmatrix} 0 \\ 1 \end{pmatrix} {\otimes } \begin{pmatrix} 1 \\ 0 \end{pmatrix} \right] , \end{aligned}$$

where $\kappa _2 = \psi / (2 - \psi )$. The quantity $\kappa _2$ determines the pairwise correlations present in this state, since $\big ( \underline{\Omega }^{\mathrm {T}} \otimes \underline{\Omega }^{\mathrm {T}} \big ) \underline{A}_2 \equiv \kappa _2$. For $f \rightarrow 0$, $\kappa _2 \rightarrow 0$ and $\underline{A}_2 \rightarrow \underline{A}_1 \otimes \underline{A}_1$. With these equilibrium distributions, we may explicitly compute $\mu (t)$ and $\sigma (t)^2$ in both protocols using Eqs. (7) and (11). For the common mean, we obtain

18 $$\begin{aligned} \mu (t) = f p \, e^{-f g p r t}, \end{aligned}$$

and for the two variances, we need the various correlation functions in Eqs. (7b) and (11b). For the Hebb protocol, these are

19a $$\begin{aligned} \mathsf {E}\left[ S_1(t) S_2(t) \, | \, {+} {+} \right]&= p^2 \, e^{- (2 - f p) f g p r t} \nonumber \\&\quad + \Big [ 1 - p \, (2-p) \, e^{- (2 - f p) f g p r t} \Big ] \kappa _2, \end{aligned}$$

19b $$\begin{aligned} \mathsf {E}\left[ S_1(t) S_2(t) \, | \, {+} {\times } \right]&= \Big [ 1 - p \, e^{- (2 - f p) f g p r t} \Big ] \kappa _2, \end{aligned}$$

19c $$\begin{aligned} \mathsf {E}\left[ S_1(t) S_2(t) \, | \, {\times } {\times } \right]&= \kappa _2, \end{aligned}$$

and for the Hopfield protocol, we just set $\kappa _2 = 0$ in these equations. These results allow us to determine SNR lifetimes for simple, SU synapses. Approximating the Hopfield variance by its asymptotic form $\sigma (\infty )^2$, the single-perceptron SNR memory lifetime in the Hopfield protocol for a stochastic updater is then

20 $$\begin{aligned} \tau _{\mathrm {snr}} \approx \frac{1}{2fgpr} \log _e \frac{f^2 p^2}{\sigma _N (\infty )^2}, \end{aligned}$$

and for the population SNR lifetime $\tau _{\mathrm {pop}}$, we replace $\sigma _N(\infty )$ by $\sigma _{\mathscr {N}}(\infty ) / \sqrt{g}$, where the subscript in $\sigma _X(\infty )^2 = [f + (1-f) \zeta ^2]/X$ indicates either *N* or $\mathscr {N} = N P$.

To determine FPT lifetimes, we require $\mathsf {Prob}[ \, h \, | \, h' \, ]$ for the MIE approach, or the induced jump moments $A(h')$ and $B(h')$ for the FPE method. We relegate the derivation of $\mathsf {Prob}[ \, h \, | \, h' \, ]$ to Appendix A, where we also indicate our numerical methods for obtaining FPTs. From Appendix A, we obtain the jump moments in Eq. (5) for the FPE approach to FPTs. For both protocols we get the same first jump moment

21 $$\begin{aligned} A(h') = - \psi g h', \end{aligned}$$

and for the second jump moment, we get

22a $$\begin{aligned} B(h' \, | \, N_{\mathrm {eff}})&= \left( \frac{N_{\mathrm {eff}}}{N^2} + \frac{N - N_{\mathrm {eff}}}{N^2} \, \zeta ^2 \right) \psi (2 - \psi ) g \nonumber \\&\quad + \psi ^2 g \, (h')^2 + \frac{N_{\mathrm {eff}}(N_{\mathrm {eff}}- 1)}{N^2} \psi ^2 g \nonumber \\&\quad + 2 \, \frac{N_{\mathrm {eff}}(N - N_{\mathrm {eff}})}{N^2} \, \psi ^2 g \, \zeta \nonumber \\&\quad + \frac{(N - N_{\mathrm {eff}}) (N - N_{\mathrm {eff}}-1 )}{N^2} \psi ^2 g \, \zeta ^2, \end{aligned}$$

22b $$\begin{aligned} B(h' \, | \, N_{\mathrm {eff}})&= \left( \frac{N_{\mathrm {eff}}}{N^2} + \frac{N - N_{\mathrm {eff}}}{N^2} \, \zeta ^2 \right) \psi (2 - \psi ) g \nonumber \\&\quad + \psi ^2 g \, (h')^2, \end{aligned}$$

for the Hebb and Hopfield protocols, respectively. We have explicitly indicated the dependence of $B(h')$ on $N_{\mathrm {eff}}$, where $N_{\mathrm {eff}}$ is the number of a perceptron’s synapses that are active during the storage of $\underline{\xi }^0$. We write $B(h' \, | \, N_{\mathrm {eff}}) = \psi g B_0(N_{\mathrm {eff}}) + \psi ^2 g \, (h')^2$, where we separate out the quadratic dependence on $h'$ and it is convenient to remove an overall factor of $\psi g$ from the definition of $B_0(N_{\mathrm {eff}})$. Dropping the quadratic term from $B(h' \, | \, N_{\mathrm {eff}})$ is equivalent to considering dynamics based on the Ornstein–Uhlenbeck process (Uhlenbeck and Ornstein 1930), which we have found to be a very good approximation (Elliott 2014, 2017a, 2019), so we work with just the constant term.

For the MIE approach to FPTs, a technical difficulty as discussed in Appendix A requires us to restrict to the specific case of $\zeta = 0$ only. We use numerical methods to obtain FPT lifetimes from the MIE approach, but for small *fN*, the dynamics are dominated by $N_{\mathrm {eff}}= 1$. For $N_{\mathrm {eff}}= 1$ and $\vartheta = 0$, Eq. (4) is trivial because the only contribution to the sum involves no transition, occurring with probability $1 - {\textstyle \frac{1}{2}}\psi g$ regardless of the protocol. Writing $\sigma _{\mathrm {fpt}}^2$ as the variance in the FPT, we obtain

23 $$\begin{aligned} \text{ MIE } \quad \left\{ \begin{array}{l} \tau _{\mathrm {mfpt}} \sim {\displaystyle \frac{N (1+p)}{p g r}}, \\ \sigma ^2_{\mathrm {fpt}} \sim {\displaystyle \frac{4 N (1+p)}{p^2 f g^2 r}}, \end{array} \right. \end{aligned}$$

at leading order, for small *f* ($= g$) in both protocols. We see that $\tau _{\mathrm {mfpt}}$ scales as 1/*f* in this regime, but that $\sigma _{\mathrm {fpt}}$ scales as $1/f^{3/2}$. Although $\sigma _{\mathrm {fpt}}$ swamps $\tau _{\mathrm {mfpt}}$ for small *f*, $\tau _{\mathrm {mfpt}}$ is nevertheless robustly positive. We may use our earlier results to obtain the corresponding forms for the FPE approach to FPT lifetimes for small *f* (see Eqs. (3.29) and (3.30) in Elliott 2017a). We obtain

24 $$\begin{aligned} \text{ FPE } \quad \left\{ \begin{array}{l} \tau _{\mathrm {mfpt}} \sim {\displaystyle \frac{\log _e 2}{2 p f g r}},\\ \sigma ^2_{\mathrm {fpt}} \sim {\displaystyle \frac{\pi ^2 + 6 \log _e^2 2}{24 p^2 f^2 g^2 r}}. \end{array} \right. \end{aligned}$$

In contrast to Eq. (23), now $\tau _{\mathrm {mfpt}}$ scales as $1/f^2$ and not 1/*f*, and $\sigma _{\mathrm {fpt}}$ scales as $1/f^2$ and not $1/f^{3/2}$. Moreover, in the FPE approach, the FPT moments have lost their overall scaling with *N*. Although the forms in Eq. (24) are obtained using mean field approximations that are expected to be invalid when *fN* is small, in fact we obtain the same scaling behaviour when the expectation values are obtained by averaging properly over $h_0$ and $N_{\mathrm {eff}}$. Our simulation results, discussed in Sect. 4, agree with the behaviour in Eq. (23). Therefore, the failure of the FPE approach for small *fN* in Eq. (24) is due to the approximations intrinsic to the FPE approach itself. These include the diffusion and especially the continuum limit. For small *fN*, the system is nowhere near the continuum limit, so the scaling behaviour must be incorrect there.

### Complex synapses

We now turn to models of complex synapses that have internal states, so that $s > 1$. In such models, synapses can undergo metaplastic changes in their internal states without expressing changes in synaptic strength. We will only consider SNR lifetimes in relation to complex synapses. We have studied FPT lifetimes for filter-based synaptic plasticity for both bistate (Elliott 2017b) and multistate (Elliott 2020) synapses in a non-sparse coding context, but we have yet to consider other models of complex synapses. We therefore restrict to SNR lifetimes, but with the caveat that they are valid only in an asymptotic regime.

**Fig. 3:**
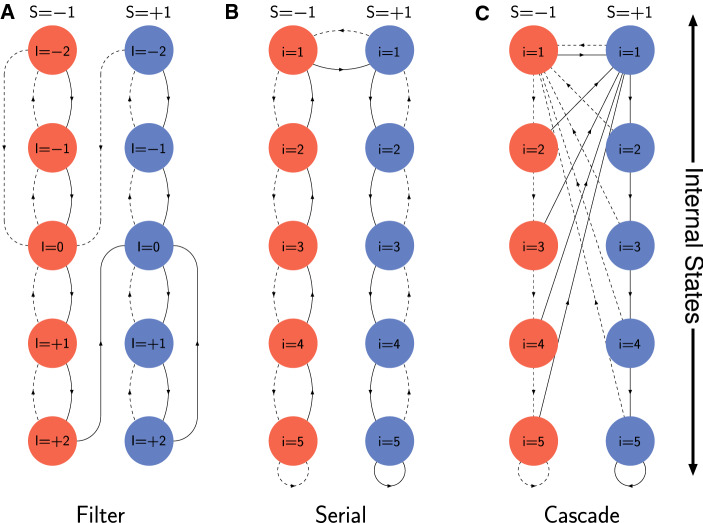
Strength and internal state transitions for various models of complex synapses. Coloured circles indicate synaptic states, with red (respectively, blue) circles corresponding to strength $S=-1$ (respectively, $S=+1$), and the labelled numbers inside the circles identifying the particular internal states (indexed by *I* for filter states and *i* for serial and cascade states). Different internal states of the same strength state are organised in the same vertical column, while different strength states correspond to different columns. Solid (respectively, dashed) lines between states show transitions caused by potentiating (respectively, depressing) induction signals, with arrows indicating the direction of the transition. Loops to and from the same state indicate no transition. Three different models are shown, as labelled, corresponding to a $\Theta = 3$ filter model (**A**), and $s = 5$ serial (**B**) and cascade (**C**) synapse models. For the filter and serial synapse models, given the presence of an induction signal of the correct type, the transition probabilities are unity. For the cascade model, the transition probabilities are as discussed in the main text

We have discussed filter-based models of synaptic plasticity at length elsewhere (Elliott 2008; Elliott and Lagogiannis 2009, 2012; Elliott 2016b), so we only briefly summarise them here. Synapses are proposed to implement a form of low-pass filtering by integrating plasticity induction signals in an internal filter state. Synapses then filter out high-frequency noise in their induction signals and pass only low-frequency trends, rendering them less susceptible to changes in strength due to fluctuations in their inputs. Potentiating (respectively, depressing) induction signals increment (respectively, decrement) the filter state, with synaptic plasticity being expressed (if possible) only when the filter reaches an upper (respectively, lower) threshold. For symmetric potentiation and depression processes, we may take these thresholds to be $\pm \Theta$. The filter can occupy the $2 \Theta -1$ states $-(\Theta -1), \ldots , +(\Theta -1)$, with the thresholds $\pm \Theta$ not being occupiable states. Several variant filter models are distinguishable by their different dynamics upon reaching threshold (Elliott 2016b), but we consider only the simplest of them here. In the simplest model, the filter always resets to the zero filter state upon reaching threshold, regardless of its strength state and regardless of the type of plasticity induction signal. This filter generalises to any multistate synapse. If the synapse is saturated at its upper (respectively, lower) strength state and reaches its upper (respectively, lower) filter threshold upon receipt of a potentiating (respectively, depressing) induction signal, the filter resets to zero despite the fact that it cannot increment (respectively, decrement) its strength. The transitions for this filter for the case of $\Theta = 3$ are illustrated in Fig. 3A. Although for clarity we have shown all permitted transitions between all filter and strength states, we stress that each synapse possesses only a single synaptic filter: the filter is not duplicated for each strength state. Transitions in filter state occur independently of strength state. Nevertheless, to describe transitions in the joint strength and filter state, we require $2(2 \Theta -1) \times 2(2 \Theta -1)$ matrices, so $s = 2 \Theta - 1$, although the number of required physical states for filter-based synapses is just $2 \Theta - 1$ for the filter states themselves, and an additional, binary-valued variable for the bistate strength, so a total of $2 \Theta$ states.

We state without derivation the result for $\mu (t)$ in this filter model:

25 $$\begin{aligned} \mu _{\mathrm {fil}}(t)&= \frac{f}{\Theta ^3} \sum _{l=0}^{\Theta -1} \cot ^2 {\textstyle \frac{(2l+1) \pi }{4 \Theta }} \, e^{- f g r t \left[ 1 - \cos \frac{(2l+1) \pi }{2 \Theta } \right] } \nonumber \\&\quad - \frac{4f}{\Theta ^3} \sum _{l=0}^{\big \lfloor \frac{\Theta -1}{2}\big \rfloor } \cot ^2 {\textstyle \frac{(2l+1) \pi }{2\Theta }} \, e^{- f g r t \left[ 1 - \cos \frac{(2l+1) \pi }{\Theta } \right] }, \end{aligned}$$

where $\lfloor \cdot \rfloor$ denotes the floor function. This expression is obtained from Eq. (4.24) in Elliott (2016b) just by multiplying by *f* and inserting a factor of *fg* into the exponents. This result is required for obtaining SNR lifetimes. The pairwise correlation functions required for $\sigma (t)^2$ are computed via numerical matrix methods using the matrices $\mathbb {M}_\pm$ for the filter model (given in Elliott (2016b) or implied by the transitions in Fig. 3A), and we also obtain the Hebb equilibrium distribution $\underline{A}_2$ by numerical methods.

To estimate SNR lifetimes in the filter model for the Hopfield protocol, we consider the slowest decaying mode in the first and second terms of Eq. (25). For non-sparse coding, it is usually enough to consider just the slowest mode in the first term, but with sparseness, both terms must be considered for a better approximation. For $\Theta$ large enough, we then have

26 $$\begin{aligned} \mu _{\mathrm {fil}}(t) \approx \frac{16 f}{\pi ^2 \Theta } \left( e^{-\pi ^2 f g r t / 8 \Theta ^2} - e^{-\pi ^2 f g r t / 2 \Theta ^2} \right) . \end{aligned}$$

Approximating the Hopfield variance by its asymptotic form $\sigma (\infty )^2$, the single-perceptron SNR memory lifetime for the filter model is then

27 $$\begin{aligned} f g r \tau ^{\mathrm {(fil)}}_{\mathrm {snr}} \approx \frac{4 \Theta ^2}{\pi ^2} \log _e \frac{256}{\pi ^4 \Theta ^2} \frac{f^2}{\sigma _N(\infty )^2} - \frac{\pi ^4 \Theta ^5}{512} \frac{\sigma _N(\infty )^3}{f^3}, \nonumber \\ \end{aligned}$$

where in deriving this expression, we have regarded the second term as a correction to the first term, with the first term arising purely from the first term in Eq. (26). To obtain the population SNR memory lifetime $\tau ^{\mathrm {(fil)}}_{\mathrm {pop}}$, we again just replace $\sigma _N(\infty )$ by $\sigma _{\mathscr {N}}(\infty )/\sqrt{g}$ in Eq. (27).

We also consider the serial synapse model (Leibold and Kempter 2008; Rubin and Fusi 2007). In this model, a synapse performs a symmetric, unbiased, one-step random walk on a set of 2*s* states between reflecting boundaries. The first (respectively, second) group of *s* states are identified as corresponding to strength $-1$ (respectively, $+1$). For each strength state, there are thus *s* metastates. If a synapse has strength $-1$ (respectively, $+1$) and experiences a sequence of depressing (respectively, potentiating) induction signals, then it is pushed into progressively higher metastates. However, the synapse can only change strength when in the lowest, $i=1$ metastate. The transitions are illustrated in Fig. 3b. The transition matrices $\mathbb {M}_\pm$ are just

28a $$\begin{aligned} \mathbb {M}_+&= \mathrm {diag}\{ \overbrace{0 , \ldots , 0}^{2s-1} , 1 \} + \mathrm {diag}_l \{ \overbrace{1, \ldots , 1}^{2s-1} \}, \end{aligned}$$

28b $$\begin{aligned} \mathbb {M}_-&= \mathrm {diag}\{ 1, \underbrace{0, \ldots , 0}_{2s-1} \} + \mathrm {diag}_u \{ \underbrace{1, \ldots , 1}_{2s-1} \}, \end{aligned}$$

where $\mathrm {diag}_u$ and $\mathrm {diag}_l$ denote the upper and lower diagonals, respectively. The eigen-decomposition of $\mathbb {M} = \frac{1}{2}\left( \mathbb {M}_+ {+} \mathbb {M}_- \right)$ is standard (cf. Elliott 2016a, for the eigen-decomposition of the similar matrix $\mathbb {C}$ there), so we can directly evaluate $\mu (t) = f \, \underline{\Omega }^{\mathrm {T}} e^{\left( \mathbb {M} - \mathbb {I} \right) f g r t} \, \mathbb {M}_+ \underline{A}_1$, where $\underline{A}_1^{\mathrm {T}} = \left( \, \underline{1}^{\mathrm {T}} \; | \; \underline{1}^{\mathrm {T}} \right) /(2s)$. We obtain

29 $$\begin{aligned} \mu _{\mathrm {ser}}(t) = \frac{f}{s^2} \sum _{l=0}^{s-1} (-1)^l \cot {\textstyle \frac{(2l+1)\pi }{4s}} \, e^{ - f g r t \left[ 1 - \cos \frac{(2l+1)\pi }{2s} \right] }. \end{aligned}$$

For the Hebb protocol, we again use numerical matrix methods to obtain $\underline{A}_2$. To estimate SNR lifetimes for the Hopfield protocol, it is sufficient to consider just the slowest decaying term in Eq. (29), giving

30 $$\begin{aligned} \mu _{\mathrm {ser}}(t) \approx \frac{4f}{\pi s} e^{-\pi ^2 f g r t/8 s^2}, \end{aligned}$$

for *s* large enough, and hence

31 $$\begin{aligned} f g r \tau ^{\mathrm {ser}}_{\mathrm {snr}} \approx \frac{4s^2}{\pi ^2} \log _e \frac{16}{\pi ^2 s^2} \frac{f^2}{\sigma _N(\infty )^2}, \end{aligned}$$

as the required approximation, with $\tau ^{\mathrm {ser}}_{\mathrm {pop}}$ obtained in the usual way.

In the cascade model of synaptic plasticity (Fusi et al. 2005), there are also 2*s* metalevels, *s* for each bistate strength state, but unlike the serial synapse model, a potentiating (respectively, depressing) induction signal for a synapse with strength $-1$ (respectively, $+1$) in metastate *i* can with probability $2^{1-i}$ (or $2^{2-i}$ for $i = s$) cause the synapse to change strength and return to metastate $i=1$. The same probabilities govern transitions to higher metastates. The transitions are illustrated in Fig. 3C. The cascade model essentially constitutes a tower of stochastic updaters that progressively render the synapse less labile. We have extensively analysed the cascade model elsewhere (Elliott and Lagogiannis 2012) and compared its memory performance to filter-based synapses, which outperform the cascade model in almost all biologically relevant regions of parameter space (Elliott 2016b). It is possible to obtain analytical results for the Laplace transform of the mean dynamics in the cascade model (Elliott and Lagogiannis 2012), but here we use numerical matrix methods. Rubin and Fusi (2007) give a formula for the SNR based on finding a fit to numerical results. The implied formula for the mean is

32 $$\begin{aligned} \mu _{\mathrm {cas}}(t) \approx \frac{14f}{5 s} \frac{e^{-f g r t / 2^{s-2}}}{1+ f g rt}. \end{aligned}$$

Taking the asymptotic variance $\sigma (\infty )^2$ in the Hopfield protocol, we can then use the expression $\mu _{\mathrm {cas}}(t) / \sigma _N(\infty )$ for the SNR. This still cannot be solved analytically for the SNR lifetime $\tau ^{\mathrm {cas}}_{\mathrm {snr}}$ (or the population form $\tau ^{\mathrm {cas}}_{\mathrm {pop}}$), but we can use it to obtain numerical solutions that can be compared to results obtained from exact matrix methods.

A serial or cascade synapse possesses 2*s* states, with each set of *s* metalevels duplicated for each strength. Metalevel *i* for strength $-1$ cannot be identified with metalevel *i* for strength $+1$ because the transitions induced by plasticity induction signals are in opposite directions. This is in contrast to the filter model, in which the filter transitions are independent of the strength state. Serial and cascade synapses therefore possess fully 2*s* physical states characterising the state of a synapse, while a filter synapse possesses $2 \Theta$ physical states and not $2 (2 \Theta - 1)$ states. Hence, we may directly compare the performance of a filter synapse with threshold $\Theta$ to a serial or cascade synapse with a total of 2*s* metastates, or *s* metastates per strength state.

## Results

We now turn to a discussion of our results, comparing and contrasting the various models of synaptic plasticity considered above, for the Hebb and Hopfield protocols. For simplicity we consider simulation results only for SU synapses, to confirm and validate our analytical results. Simulations are run according to protocols discussed extensively elsewhere (see, for example, Elliott and Lagogiannis 2012; Elliott 2014), but modified to allow for sparse coding. We first consider single-perceptron memory lifetimes and then population memory lifetimes.

### Single-perceptron memory lifetimes

**Fig. 4:**
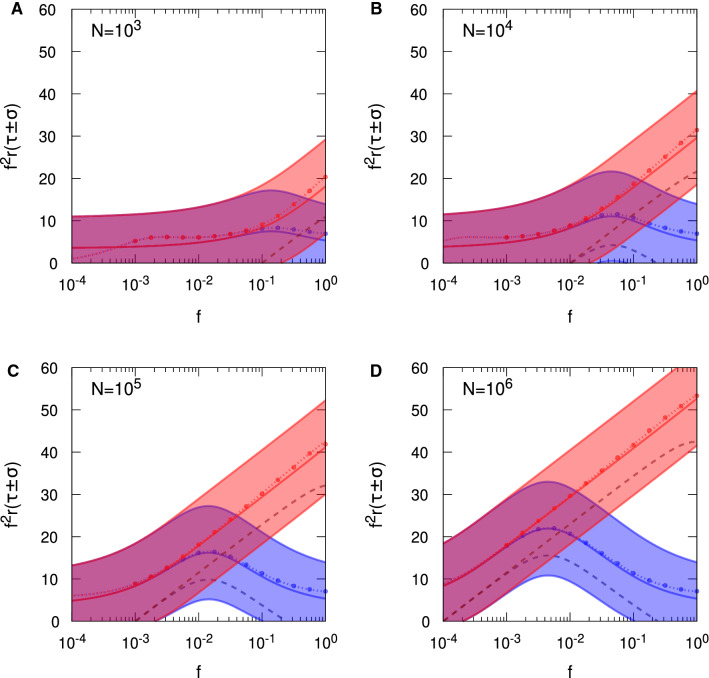
Convergence of Hebb and Hopfield protocol results for stochastic updater synapses in the limit of sparse coding. Scaled single-perceptron memory lifetimes are shown as a function of sparseness, *f*. Results in red (respectively, blue) correspond to the Hopfield (respectively, Hebb) protocol. Shaded regions indicate $f^2 r ( \tau _{\mathrm {mfpt}} \pm \sigma _{\mathrm {fpt}} )$ (with the central solid line showing $f^2 r \tau _{\mathrm {mfpt}}$) computed using the FPE approach to FPTs, so that we show the (scaled) MFPT $\tau _{\mathrm {mfpt}}$ surrounded by the one standard deviation region around it, governed by $\sigma _{\mathrm {fpt}}$. Short-dashed lines show $f^2 r \tau _{\mathrm {mfpt}}$ obtained using the exact, MIE approach to FPTs. Circular data points correspond to results from simulation, for $f \ge 10^{-3}$. Long-dashed lines show results for $f^2 r \tau _{\mathrm {snr}}$; $r \tau _{\mathrm {snr}} = 0$ for the Hebb protocol over the whole range of *f* in panel **A**. The value of *N* is indicated in each panel. In all panels, $p = 1/10$, $\zeta = 0$ and $\vartheta = 0$

In Fig. 4, we show results for memory lifetimes for SU synapses with no spontaneous activity, $\zeta = 0$, comparing the Hopfield and Hebb protocols. We consider both FPT and SNR lifetimes, and for FPT lifetimes, we show results for both the FPE and MIE approaches. Simulation results are also shown, although only for $f \ge 10^{-3}$: for smaller values it becomes increasingly difficult to obtain enough statistics for decent averaging due to the longer simulation run times. We select an update probability of $p=1/10$, which is our standard choice of *p* in earlier work (see, for example, Elliott 2014). From Eq. (24), $r \tau _{\mathrm {mfpt}}$ and $r \sigma _{\mathrm {fpt}}$ are expected to scale as $1/f^2$ for small *f* for the FPE approach, so we remove this scaling by multiplying by $f^2$, which in this figure affords greater clarity and resolution.

Above we showed that the Hopfield and Hebb protocols must coincide for $f \lessapprox 1/\sqrt{N}$. For the various choices of *N* used in Fig. 4, we see this convergence of both protocols’ results, becoming indistinguishable for *f* below $1/\sqrt{N}$, for all forms of memory lifetime. Focusing first on $r \tau _{\mathrm {mfpt}}$ from the FPE approach, for smaller *N* we clearly see that $f^2 r \tau _{\mathrm {mfpt}}$ asymptotes to a common, *N*-independent constant as *f* becomes small; we would see the same behaviour for larger *N* too, but would need to take smaller values of *f* than those used in this figure. We also see that $f^2 r \tau _{\mathrm {mfpt}}$ from the MIE approach tracks that from the FPE approach quite closely, and indeed for intermediate values of *f* and smaller choices of *N*, it plateaus, so that $r \tau _{\mathrm {mfpt}}$ scales as $1/f^2$ in this regime. However, for $N=10^3$ and $f \lessapprox 10^{-3}$, we clearly see the MIE $f^2 r \tau _{\mathrm {mfpt}}$ turn downwards and approach zero as *f* decreases. This behaviour is consistent with the derived form of the exact scaling behaviour in Eq. (23), in which $r \tau _{\mathrm {mfpt}} \propto 1/f$ for small *f*. We also just see this change for $N=10^4$ for *f* close to $10^{-4}$, but for larger *N* we would need to take *f* smaller to see the 1/*f* scaling of the exact form of $r \tau _{\mathrm {mfpt}}$. Our simulation results agree with the results from the MIE approach, validating both. Although we do not take *f* small enough to see the switch to 1/*f* scaling for $N=10^3$ in Fig. 4A, we nevertheless do clearly see the start of the down-turn at $f=10^{-3}$.

For $f > 1/\sqrt{N}$ in Fig. 4, we see very significant differences between the Hebb and Hopfield protocols. While for the Hopfield protocol $f^2 r \tau _{\mathrm {mfpt}}$ grows like $\log _e N$ for *fN* large enough, this is not the case for the Hebb protocol. For *f* in the region of unity, $f^2 r \tau _{\mathrm {mfpt}}$ is roughly speaking independent of *N*. This means that the dynamics are dominated by the correlations between pairs of synapses’ strength in the Hebb protocol. For $f=1$, we obtain $r \tau _{\mathrm {mfpt}} \approx 5.34$ and 5.35 for $N=10^3$ and $N=10^6$, respectively, from the FPE approach. (The corresponding values from the MIE approach are 6.64 and 6.79, respectively.) In the regime of *f* not too far from unity, memory lifetimes in the Hebb protocol are therefore significantly reduced by the synaptic correlations induced by this protocol, where the influence of these correlations cannot be removed by increasing *N*.

We see that $r \tau _{\mathrm {mfpt}}$ is robustly positive in Fig. 4 for all choices of *N* over the whole range of displayed *f*, and it remains so for small *f* because of the discussed scaling behaviour. However, looking at the one standard deviation region around $r \tau _{\mathrm {mfpt}}$, it is clear that in some regimes for *f*, there can be high variability in FPT memory lifetimes. For the Hopfield protocol, this regime of high variability occurs for small *f* (where what is “small” *f* depends on *N*), while in the Hebb protocol, there is an additional regime for *f* close to unity. High variability does not mean that memories cannot be stored: $r \tau _{\mathrm {mfpt}}$ is always robustly positive. Rather, high variability simply means that some memories are stored strongly while others are stored weakly or not at all.

Turning to a consideration of $r \tau _{\mathrm {snr}}$, we see from Fig. 4 that $r \tau _{\mathrm {snr}}$ exists (i.e. $r \tau _{\mathrm {snr}} > 0$) in precisely those regions of low variability in FPT lifetimes. Indeed, the results for $r \tau _{\mathrm {snr}}$ track quite closely those for $r (\tau _{\mathrm {mfpt}} - \sigma _{\mathrm {fpt}})$ over some range of *f*, and deviate from it elsewhere. We have shown in a non-sparse coding context that FPT and SNR lifetimes for simple synapses essentially coincide (up to additive constants) in the regime in which the distribution of $h_0$ is tightly concentrated around its supra-threshold mean (Elliott 2017a). For the specific case of $\vartheta = 0$, as here, we showed that if we can write the initial variance $\sigma (0)^2$ in the form $\sigma (0)^2 \approx B_0(N)/2$, then the parameter $\mu ' \equiv \mu (0) \sqrt{2/B_0(N)}$ must be large enough, which means $\mu ' \gtrapprox 2$ (Elliott 2017a). We then have that $\mu ' \approx \mu (0) / \sigma (0) \gtrapprox 2$, which is just a condition on the initial SNR.^2^ Using the pre-averaged form $\langle B_0(N_{\mathrm {eff}}) \rangle _{N_{\mathrm {eff}}}$ (see Appendix A), this condition reduces to $4/(p^2 N) \lessapprox f$ in the Hopfield protocol for $\zeta = 0$. For the Hebb protocol, the limit of large *N* with *p* not too close to unity additionally satisfies the requirement on $\sigma (0)^2$, giving the upper bound $f \lessapprox p / 2$ for $\zeta = 0$. In the Hebb protocol, we therefore have the interval $4/(p^2 N) \lessapprox f \lessapprox p / 2$ for equivalence of SNR and FPT memory lifetimes, for $\zeta = 0$. (We must have $N \gtrapprox 8/p^3$ for this interval to exist.) With $p = 1/10$ in Fig. 4, these conditions are $400/N \lessapprox f$ and $400/N \lessapprox f \lessapprox 0.05$ for the Hopfield and Hebb protocols, respectively. For $400/N \lessapprox f$ in both protocols (except for the Hebb protocol for $N=10^3$, where the bounding range of *f* is invalid), we do indeed see that the FPE results for $f^2 r \tau _{\mathrm {mfpt}}$ and those for $f^2 r \tau _{\mathrm {snr}}$ run essentially parallel to each other, but that for $f < 400/N$, $f^2 r \tau _{\mathrm {snr}}$ peels away from $f^2 r \tau _{\mathrm {mfpt}}$. The same is true for the Hebb protocol for $N > 10^3$: as *f* increases above 0.05, $f^2 r \tau _{\mathrm {snr}}$ also peels away from $f^2 r \tau _{\mathrm {mfpt}}$. Thus, these two estimates for the two protocols appear to capture well the region of *f* for which $r \tau _{\mathrm {snr}}$ is a reliable indicator of memory longevity. SNR lifetimes are therefore acceptable surrogates for FPT lifetimes when the latter are subject to low variability, but outside these regions SNR lifetimes fail to capture the possibility of memory storage, albeit with high variability. Importantly, the requirement that $f \gtrapprox 4/(p^2 N)$ in both protocols means that the SNR approach cannot be extended to very small or just small *f*, because such values violate the asymptotic regime. Essentially, then, the SNR approach cannot probe the very sparse coding regime in either protocol.

For the Hopfield protocol, Eq. (20) is just

33 $$\begin{aligned} r \tau _{\mathrm {snr}} \approx \frac{1}{2 f^2 p} \log _e \frac{N f^2 p^2}{f + (1-f) \zeta ^2}. \end{aligned}$$

With $\zeta = 0$, we require $f>1/(p^2 N)$ for $r \tau _{\mathrm {snr}} > 0$, and we see precisely these threshold values for the different choices of *N* in Fig. 4. Alternatively, we require $N>1/(fp^2)$ for memories to be stored according to the SNR criterion. However, these conditions do not carry over to FPT memory lifetimes: we need neither a minimum *N* nor a minimum *f* for $r \tau _{\mathrm {mfpt}} > 0$, because it is always positive. This failure of SNR conditions to carry over to the FPT case also applies to any optimality conditions derived from $r \tau _{\mathrm {snr}}$. From Eq. (33) with $\zeta = 0$, we may find that value of *f*, $f^{\mathrm {opt}}$, that maximises $r \tau _{\mathrm {snr}}$, giving rise to $r \tau _{\mathrm {snr}}^{\mathrm {opt}}$, with the result that $f^{\mathrm {opt}} = \sqrt{e}/(p^2 N)$. The same value essentially applies to the Hebb protocol, albeit with complicated corrections. However, for the validity of the SNR results, both protocols require $f \gtrapprox 4/(p^2 N)$. If the SNR optimality condition is valid, then it must satisfy $f^{\mathrm {opt}} = \sqrt{e}/(p^2 N) \gtrapprox 4/(p^2 N)$, or $\sqrt{e} \gtrapprox 4$. This is clearly false, and hence the SNR optimality condition for *f* is spurious, because at $f = f^{\mathrm {opt}}$, the asymptotic validity condition is violated. In fact, we may essentially take *f* as small as we like and $r \tau _{\mathrm {mfpt}}$ will continue to grow, albeit with increasing variability in the FPT lifetimes. Thus, although we will shortly consider optimality conditions for SNR memory lifetimes with complex synapses, these conditions must be viewed with extreme caution.

**Fig. 5:**
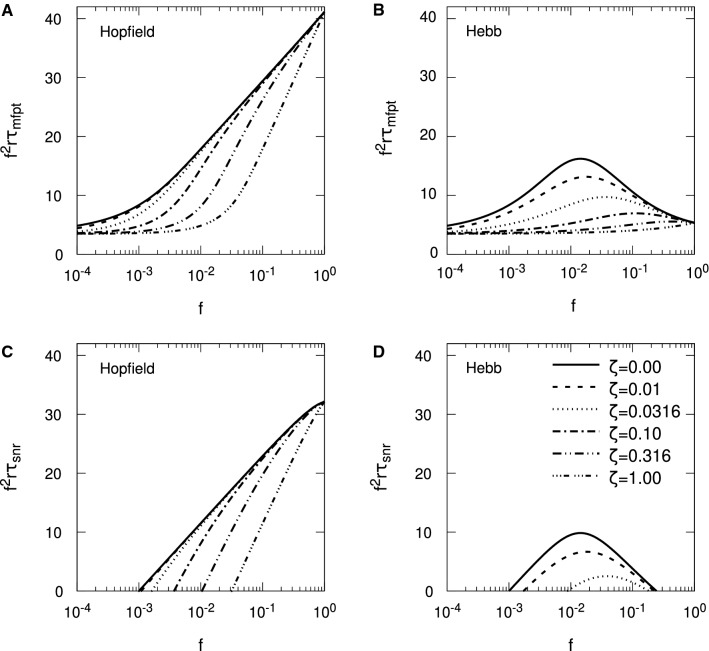
Impact of spontaneous activity on stochastic updater single-perceptron memory lifetimes. Results are shown for $f^2 r \tau _{\mathrm {mfpt}}$ (from the FPE approach) and $f^2 r \tau _{\mathrm {snr}}$ for both the Hopfield and Hebb protocols, as indicated in the different panels. Different line styles correspond to different levels of spontaneous activity, $\zeta$, as indicated in the common legend in panel **D**. Some lines style are absent in panel **D** because there is no corresponding $r \tau _{\mathrm {snr}} > 0$. In all panels we take $N = 10^5$, with $p = 1/10$ and $\vartheta = 0$ in all cases

Figure 4 considers only the case of exactly zero spontaneous activity, $\zeta = 0$. In Fig. 5, we examine the impact of spontaneous activity on SU memory lifetimes. We show only the case of $N = 10^5$ to avoid unnecessary clutter, but the results are qualitatively similar for other choices of *N*. In the Hopfield protocol, $\zeta$ appears only through a quadratic term in $B(h')$ or $\sigma (t)^2$, while in the Hebb protocol, $\zeta$ also appears through a linear term. This difference makes the Hebb protocol much more sensitive to spontaneous activity than the Hopfield protocol, and we see this explicitly in Fig. 5. In the Hopfield protocol, the asymptotic variance takes the form $\sigma (\infty )^2 = [ f + (1-f) \zeta ^2 ]/N$, so $\zeta$ exerts a significant influence on memory lifetimes only for $f \lessapprox \zeta ^2$. We therefore only start to see a divergence of memory lifetimes from those for $\zeta = 0$ at around $f \approx \zeta ^2$, and this is confirmed in the figure. However, as *f* is taken small, the dependence of $r \tau _{\mathrm {mfpt}}$ (from the FPE) on $\zeta$ is lost (just as its dependence on *N* is lost), so that for very small *f*, $\zeta$ does not affect (FPE) FPT lifetimes, neither their means nor their variances. This is because for small *f*, the scaling results in Eq. (24) depend only on the *A* and not the *B* jump moment, so they depend only on drift and not diffusion. However, $\zeta$ appears only through the diffusion term. In contrast to the Hopfield protocol, even a choice of $\zeta = 0.01$ induces a large reduction in memory lifetimes in the Hebb protocol, at least away from the small *f* regime. For small *f*, the Hebb and Hopfield protocols coincide, so we observe the same loss of dependence on $\zeta$ in (FPE) FPT lifetimes in the Hebb protocol. However, away from the small *f* regime, the linear term in $\zeta$ in *B* or $\sigma (t)^2$ significantly impacts memory lifetimes.

Examining Eq. (33) for the Hopfield protocol, for $\zeta = 0$, we have just $f^1$ in the logarithm, while for $\zeta = 1$, we have $f^2$. Roughly speaking, for intermediate values of $\zeta$, the effective power of *f* switches rapidly from one to two in the vicinity of $f = \zeta ^2$. This switching can be seen clearly in Fig. 5, where as *f* decreases, $f^2 r \tau _{\mathrm {snr}}$ (and also $f^2 r \tau _{\mathrm {mfpt}}$) tracks closely the form for $\zeta = 0$, until it rapidly peels away, following a different power. Although it is still clearly the case that optimality conditions obtained from $r \tau _{\mathrm {snr}}$ are invalid, it is nevertheless worth examining $f^{\mathrm {opt}}$. For $\zeta = 0$, we again obtain $f^{\mathrm {opt}} = \sqrt{e}/(p^2 N)$, but for $\zeta = 1$, we instead obtain $f^{\mathrm {opt}} = \sqrt{e}/(p^2 N)^{1/2}$, so that the *N*-dependence changes. The corresponding optimal lifetimes are $r \tau _{\mathrm {snr}}^{\mathrm {opt}} = p^3 N^2 / (4 e)$ for $\zeta = 0$ and $r \tau _{\mathrm {snr}}^{\mathrm {opt}} = p N / (2 e)$ for $\zeta = 1$. Of course, we see explicitly in Fig. 5 that these SNR-derived optimal values of *f* and thus maximum possible SNR lifetimes are invalid, but SNR lifetimes do at least indicate when FPT lifetimes are subject to lower variability and when they are subject to higher variability.

Considering $\zeta = 1$ is of course biologically meaningless, as then there is no distinction between spontaneous and evoked electrical activity levels. However, taking either $\zeta = 0$ or $\zeta = 1$ allows explicit optimality results to be obtained for these two cases, while such results are not available for intermediate values of $\zeta$. As just indicated, empirically we observe a very rapid switching in dynamics in the vicinity of $\zeta = \sqrt{f}$, with the explicit results for $\zeta =0$ and $\zeta =1$ therefore indicating the general behaviour prior to and after, respectively, this switching. When we give results for $\zeta = 1$, we therefore do so with this understanding: that the limit is biologically meaningless, but that it nevertheless indicates the general behaviour for $\zeta$ in excess of around $\sqrt{f}$.

**Fig. 6:**
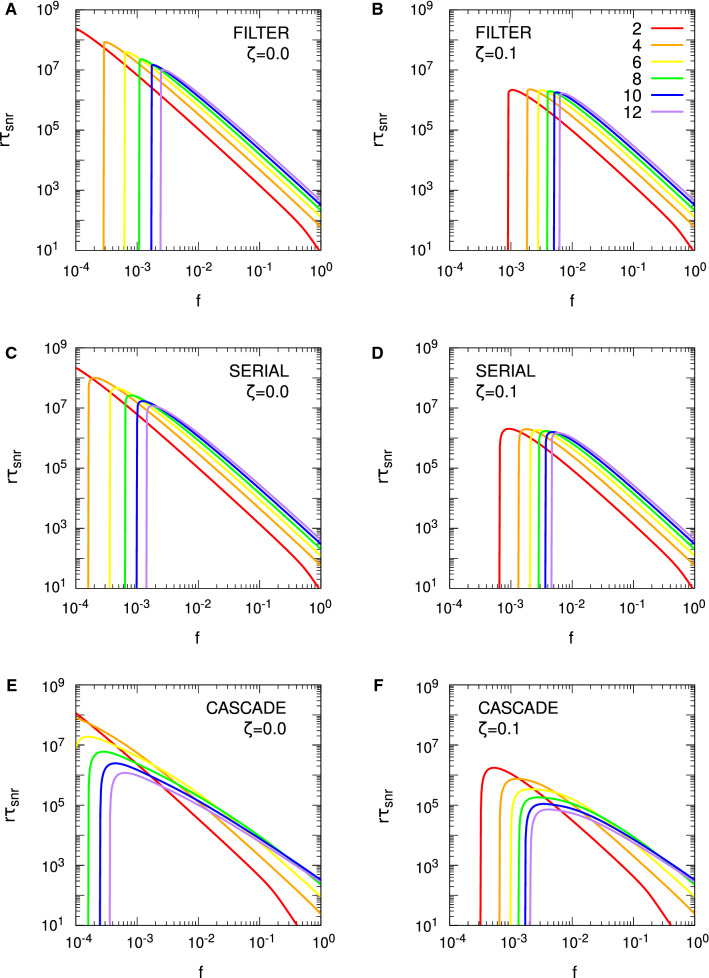
Spontaneous activity reduces single-perceptron memory lifetimes and limits sparseness in complex synapse models: Hopfield protocol. Single-perceptron SNR memory lifetimes are shown for different complex models of synaptic plasticity under the Hopfield protocol, as a function of sparseness, *f*. Each panel shows results for the indicated model and choice of spontaneous activity, either $\zeta = 0$ or $\zeta = 0.1$. Results are shown for $\Theta$ or *s* ranging from 2 to 12 in increments of 2, with the particular choice identified by the line colour described by the common legend in panel **B**. In all cases, $N = 10^5$

**Fig. 7:**
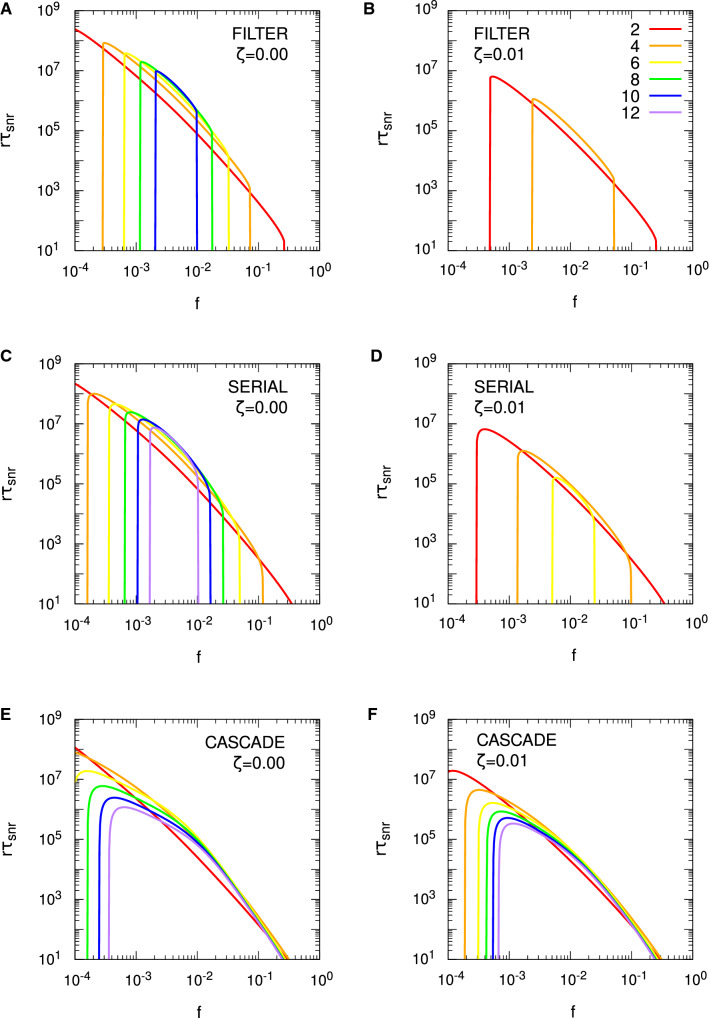
Spontaneous activity reduces single-perceptron memory lifetimes and limits sparseness in complex synapse models: Hebb protocol. The format of this figure is identical to Fig. 6, except that it shows results for the Hebb protocol, and in the right-hand panels we use a smaller value $\zeta = 0.01$. Some lines of specific colour are absent in some graphs because there is no corresponding $r \tau _{\mathrm {snr}} > 0$. In all cases, $N = 10^5$

We now turn to complex models of synaptic plasticity, considering only SNR lifetimes. In Figs. 6 and 7, we plot SNR lifetimes against sparseness, *f*, for the three complex models discussed above, for both zero and nonzero spontaneous firing rates, and for the Hopfield (Fig. 6) and Hebb (Fig. 7) protocols. All results are obtained by numerical matrix methods to solve the SNR equation $\mu (\tau _{\mathrm {snr}}) = \sigma (\tau _{\mathrm {snr}})$, where the standard deviation $\sigma (t)$ is computed fully rather than via just its asymptotic form $\sigma (\infty )$.

For the Hopfield protocol in Fig. 6 we see in all cases and for all choices of parameters an onset of SNR lifetimes for a minimum, threshold value of *f*, the rapid attainment of a peak or optimal value of $r \tau _{\mathrm {snr}}$, followed by a steady fall in lifetimes as *f* increases further. For all complex models, this onset of SNR lifetimes occurs for increasingly large values of *f* as $\Theta$ or *s* increases. At least for the parameter ranges in this figure, in the filter and serial models, for a given choice of *f*, increasing $\Theta$ or *s* increases $r \tau _{\mathrm {snr}}$, although as the number of internal states continues to increase, ultimately $r \tau _{\mathrm {snr}}$ will start to fall. In the case of the cascade model, however, the dependence of $r \tau _{\mathrm {snr}}$ on *s* for fixed *f* is not as simple as for the other complex models. We note that for all models in this figure, the optimal values of $r \tau _{\mathrm {snr}}$ decrease for increasing $\Theta$ or *s*, at least for $\zeta = 0$. However, when we increase the spontaneous activity to $\zeta = 0.1$, the optimal values lose most of their dependence on $\Theta$ or *s* in the filter and serial models, although not in the cascade model. This loss of dependence on $\Theta$ or *s* is strongly *N*- and $\zeta$-dependent. For $N=10^3$, we must take $\zeta$ close to unity before this loss of dependence is noticeable, while for $N=10^6$, even $\zeta =10^{-3/2} \approx 0.0316$ is sufficient.

For the Hebb protocol, in Fig. 7, for smaller *f* we obtain essentially the same results as for the Hopfield protocol because these two protocols must coincide for $f \lessapprox 1/\sqrt{N}$, regardless of the model of synaptic plasticity. However, for larger *f*, the synaptic correlation terms induced by the Hebb protocol again significantly impact SNR memory lifetimes, with the impact being greater for larger $\Theta$ or *s* in the filter and serial models. Thus, as with SU synapses under the Hebb protocol, SNR lifetimes exist only in some interval of *f* (below $f=1$), with this interval shrinking and disappearing as the number of internal states increases (or as *p* decreases for SU synapses). These dynamics dramatically limit the number of internal states that give rise to positive SNR lifetimes. Nevertheless, as $\Theta$ or *s* increases, $r \tau _{\mathrm {snr}}$ in general increases, at least until the permissible range of *f* becomes very small and then disappears entirely. For the cascade model, however, the upper limit on *f* is roughly speaking independent of *s*, but we also see that in general, as *s* increases, $r \tau _{\mathrm {snr}}$ decreases for fixed *f*. This relative insensitivity of the upper limit of the permissible range of *f* to *s* in the cascade model occurs because the cascade model has different metastates with different update probabilities, with some synapses residing in the lower metastates and so having larger update probabilities than those residing in higher metastates.

In the presence of spontaneous activity, we see a dramatic change in the memory lifetimes. Indeed, such is the sensitivity of the Hebb protocol to $\zeta$, especially for complex synapse models, that in contrast to Fig. 6 for the Hopfield protocol, for which we took $\zeta = 0.1$, in Fig. 7 we take $\zeta = 0.01$. Even with just 1% spontaneous activity, the filter and serial models’ number of internal states becomes severely restricted, in terms of giving rise to positive SNR lifetimes. The cascade model under the Hebb protocol is not quite so sensitive, again because of its different metastates, but a 10% level of spontaneous activity would still dramatically restrict the permissible ranges of *f* and *s*, compared to the Hopfield protocol.

**Fig. 8:**
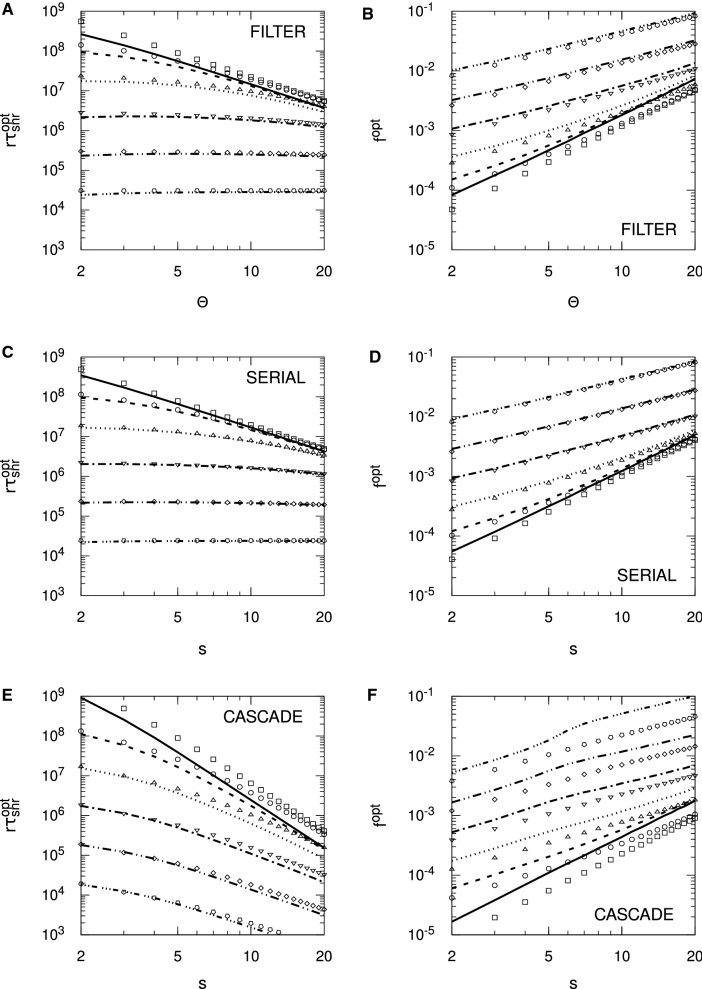
Optimal sparseness in complex synapse models for single perceptrons in the Hopfield protocol. The left-hand panels (**A**, **C**, **E**) show the optimal single-perceptron memory lifetimes $r \tau _{\mathrm {snr}}^{\mathrm {opt}}$ obtained at the corresponding optimal levels of sparseness $f^{\mathrm {opt}}$ shown in the right-hand panels (**B**, **D**, **F**), for the indicated complex models. Lines show numerical matrix results while the corresponding data points show approximate analytical results obtained as discussed in the main text. Results are shown for different values of $\zeta$, with identifying line styles corresponding to those in Fig. 5. We have set $N = 10^5$ in all panels

**Fig. 9:**
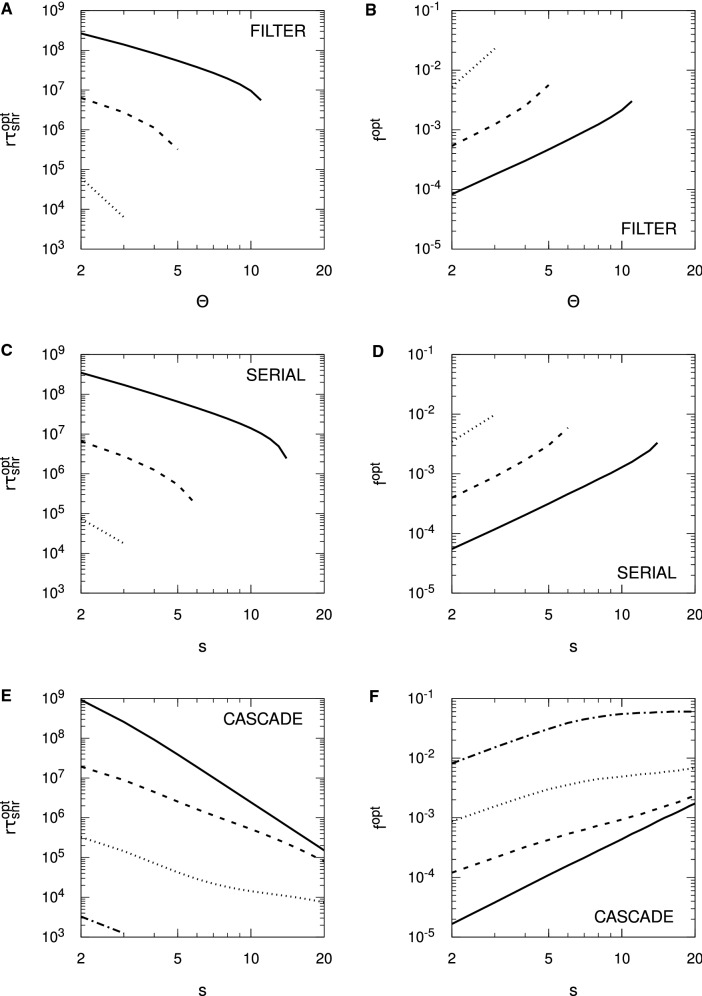
Optimal sparseness in complex synapse models for single perceptrons in the Hebb protocol. The format of this figure is essentially identical to Fig. 8, except that it shows results for the Hebb protocol. Approximate analytical results are not available for the Hebb protocol and so are not present. The termination of a line at a threshold value of $\Theta$ or *s* indicates that above that value, no choice of *f* generates $r \tau _{\mathrm {snr}} > 0$. We have set $N = 10^5$ in all panels

We quantify these observations by explicitly considering the optimal choices of the parameters *f* and either $\Theta$ or *s*, so $f^{\mathrm {opt}}$ and either $\Theta ^{\mathrm {opt}}$ or $s^{\mathrm {opt}}$, that maximise $r \tau _{\mathrm {snr}}$, giving rise to $r \tau _{\mathrm {snr}}^{\mathrm {opt}}$. In Figs. 8 and 9, we plot $f^{\mathrm {opt}}$ and $r \tau _{\mathrm {snr}}^{\mathrm {opt}}$ against $\Theta$ or *s*, for different levels of spontaneous activity, $\zeta$, for the particular choice of $N = 10^5$. Results are obtained both by numerical matrix methods and by using the approximations for $\mu (t)$ and $r \tau _{\mathrm {snr}}$ given in Sect. 3.2. For the latter, we maximise $r \tau _{\mathrm {snr}}$ as a function of *f* for fixed $\Theta$ or *s*.

For the Hopfield protocol in Fig. 8, we see that for $\zeta = 0$, $r \tau _{\mathrm {snr}}^{\mathrm {opt}}$ falls as a function of $\Theta$ or *s*. However, in the filter and serial models, as $\zeta$ increases, the fall in $r \tau _{\mathrm {snr}}^{\mathrm {opt}}$ with $\Theta$ or *s* reduces and disappears; indeed, the exact results in fact show a very slight increase in $r \tau _{\mathrm {snr}}^{\mathrm {opt}}$ with $\Theta$ or *s* for $\zeta = 1$, although this behaviour is not noticeable in Fig. 8. For the displayed choice of $N = 10^5$, we need only take $\zeta \approx 0.1$ for the filter and serial models’ $r \tau _{\mathrm {snr}}^{\mathrm {opt}}$ to be relatively insensitive to $\Theta$ or *s*. This is *N*-dependent: for $N=10^6$, even $\zeta = 0.01$ is sufficient; for $N = 10^3$, $\zeta$ needs to be quite close to unity. In contrast, for the cascade model, $r \tau _{\mathrm {snr}}^{\mathrm {opt}}$ always falls with *s* for any choice of $\zeta$, including $\zeta = 1$. The behaviour of the filter and serial models’ $r \tau _{\mathrm {snr}}^{\mathrm {opt}}$ is easy to extract from the approximate results in Sect. 3.2. Ignoring for simplicity the correction terms in Eq. (27), both filter and serial models’ $r \tau _{\mathrm {snr}}$ can be written in the form

34 $$\begin{aligned} r \tau _{\mathrm {snr}} \approx \frac{a \, q^2}{f^2} \log _e \frac{N b f^2}{q^2 [ f + (1-f) \zeta ^2]} \end{aligned}$$

(cf. Eq. (33)), where *a* and *b* are numerical constants and *q* denotes $\Theta$ or *s*. For $\zeta = 0$ we obtain $f^{\mathrm {opt}} = q^2 \sqrt{e} / (b N)$ and $r \tau _{\mathrm {snr}}^{\mathrm {opt}} \approx a b^2 N^2 / (2 e q^2)$, while for $\zeta = 1$ we obtain $f^{\mathrm {opt}} = q \sqrt{e} / \sqrt{b N}$ and $r \tau _{\mathrm {snr}}^{\mathrm {opt}} \approx a b N / e$. Therefore, $f^{\mathrm {opt}}$ scales differently with *q* and with *N* in these two cases, and for $\zeta = 0$, $r \tau _{\mathrm {snr}}^{\mathrm {opt}}$ falls as *q* increases, but for $\zeta = 1$, $r \tau _{\mathrm {snr}}^{\mathrm {opt}}$ is completely independent of *q*. Intermediate choices of $\zeta$ result in intermediate behaviours between these two extremes, and the corrections terms in Eq. (27) provide only corrections to, rather than fundamentally alter, this behaviour. We see in Fig. 8 that the numerical and approximate analytical results agree well for the filter and serial models, and that, moreover, both these models’ optimal values are very similar. Unfortunately, in the case of the cascade model, no such simple analysis, even using the fitted form for $\mu _{\mathrm {cas}}(t)$ in Eq. (32), is available to explain the fact that $r \tau _{\mathrm {snr}}^{\mathrm {opt}}$ falls with *s* for all values of $\zeta$, including $\zeta = 1$. The numerical and fitted results for $r \tau _{\mathrm {snr}}^{\mathrm {opt}}$ agree well in the cascade model, although there are quite large discrepancies in $f^{\mathrm {opt}}$ obtained from the (exact) numerical methods and the fitted expression, particularly for larger values of *s* and for $\zeta$ closer to zero than to unity. Fitting our numerical matrix results for $r \tau _{\mathrm {snr}}^{\mathrm {opt}}$ in the cascade model to power laws in *s* and *N* for large enough *s*, we find that for $\zeta = 0$, $f^{\mathrm {opt}} \sim s^2/N$ and $r \tau _{\mathrm {snr}}^{\mathrm {opt}} \sim N^2/s^4$, while for $\zeta = 1$, $f^{\mathrm {opt}} \sim s/\sqrt{N}$ and $r \tau _{\mathrm {snr}}^{\mathrm {opt}} \sim N/s^2$. While the scaling behaviour of $f^{\mathrm {opt}}$ is the same as that in the filter and serial models, the dependence of $r \tau _{\mathrm {snr}}^{\mathrm {opt}}$ on $q \equiv s$ differs in the cascade model compared to the filter and serial models.

For the Hebb protocol in Fig. 9, again the pairwise correlation structure present in $\sigma (t)^2$, and the Hebb protocol’s extreme sensitivity to even very small levels of spontaneous activity $\zeta$, have a significant impact on optimality conditions. In the filter and serial models, the permissible range of $\Theta$ or *s* is considerably reduced, so that even for $\zeta = 0.01$, *s* cannot exceed 6 in the serial synapse model, or 5 in the filter model. As *N* is reduced from the displayed value of $N = 10^5$, the permissible ranges of $\Theta$ and *s* reduce. The cascade model in the Hebb protocol is also extremely sensitive to noise, but as discussed, the different metastates’ different update probabilities somewhat ameliorate this sensitivity. Nevertheless, increasing $\zeta$ from $\zeta = 0$ to just $\zeta = 0.1$ reduces $r \tau _{\mathrm {snr}}^{\mathrm {opt}}$ by several orders of magnitude.

**Fig. 10:**
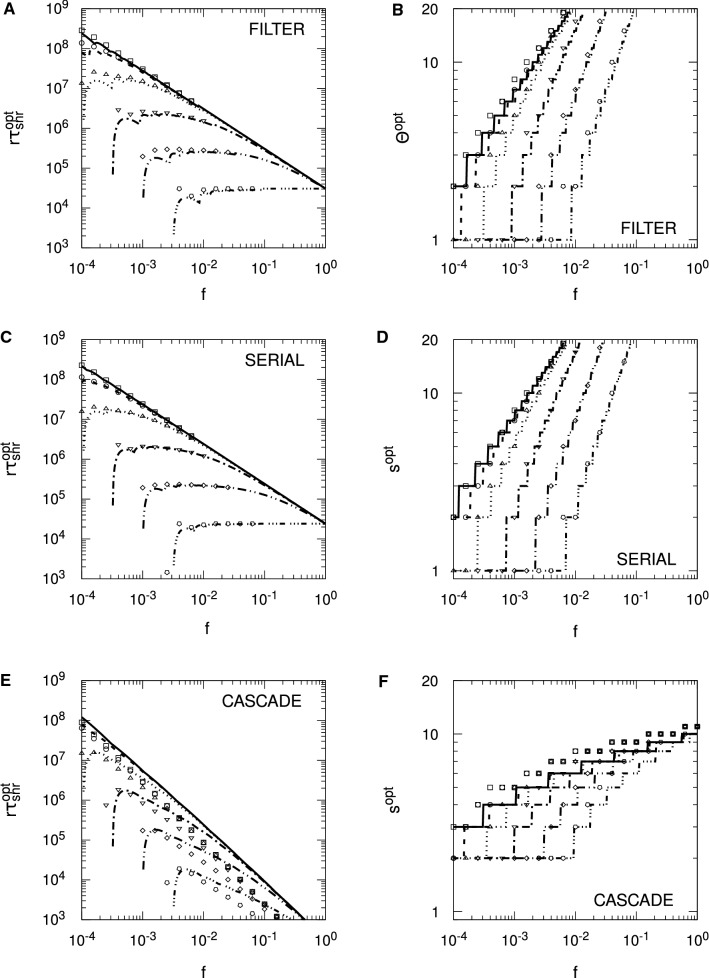
Optimal synaptic complexity in complex synapse models for single perceptrons in the Hopfield protocol. The format of this figure is very similar to Fig. 8, except that we have optimised with respect to $\Theta$ or *s* rather than *f*. In panels **A** and **C** the lines switch from numerical matrix to approximate analytical results when the corresponding values of $\Theta ^{\mathrm {opt}}$ or $s^{\mathrm {opt}}$ exceed 20 in the right-hand panels; before this transition, the lines correspond to numerical matrix results and the discrete points to approximate analytical results. We have set $N = 10^5$ in all panels

**Fig. 11:**
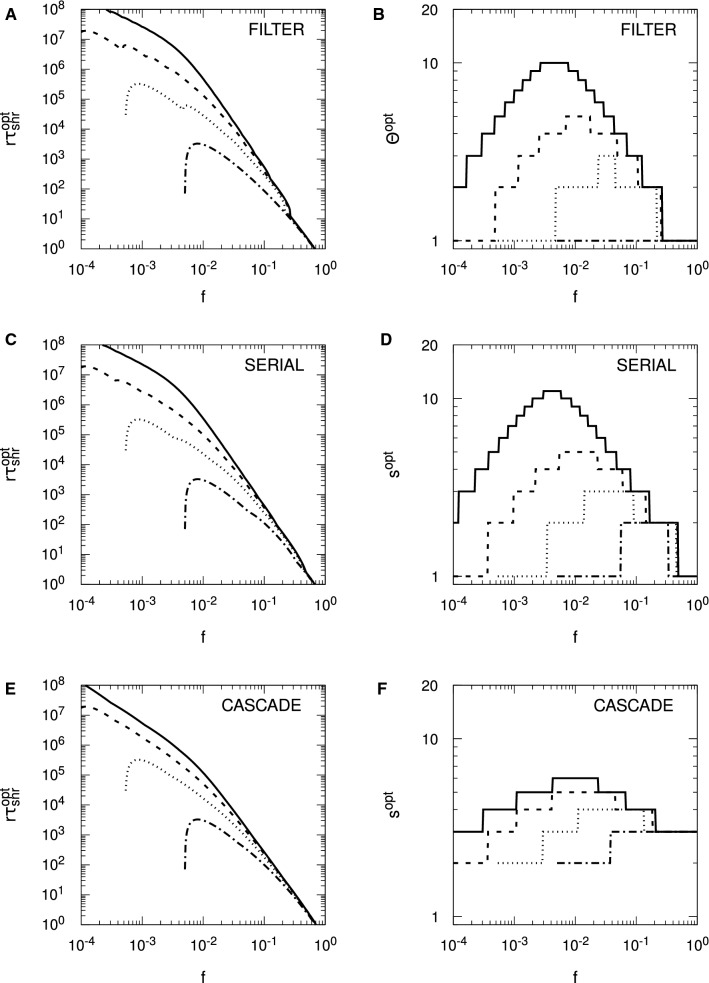
Optimal synaptic complexity in complex synapse models for single perceptrons in the Hebb protocol. The format of this figure is essentially identical to Fig. 10, except that approximate analytical results are not available for the Hebb protocol. We have set $N = 10^5$ in all panels

In Figs. 10 and 11 we instead examine $\Theta ^{\mathrm {opt}}$ or $s^{\mathrm {opt}}$ as a function of *f*, rather than *vice versa*, so that we maximise $r \tau _{\mathrm {snr}}$ with respect to $\Theta$ or *s* while holding *f* fixed. For the Hopfield protocol in Fig. 10, subject to a minimum, threshold requirement, $\Theta ^{\mathrm {opt}}$ or $s^{\mathrm {opt}}$ increases as a function of *f*, for any level of spontaneous activity $\zeta$, in all three complex models considered here. However, as $\zeta$ moves from $\zeta = 0$ to $\zeta = 1$, the functional dependence of $\Theta ^{\mathrm {opt}}$ or $s^{\mathrm {opt}}$ on *f* changes. We can derive this explicitly by again using the simple expression for $r \tau _{\mathrm {snr}}$ in Eq. (34) for the filter and serial models. The optimal value of *q* (either $\Theta$ or *s*) is

35 $$\begin{aligned} q^{\mathrm {opt}} = f \frac{\sqrt{b N}}{\sqrt{e [f + (1-f) \zeta ^2]}}. \end{aligned}$$

Thus, as *f* increases, $q^{\mathrm {opt}}$ essentially switches from linear growth in *f* to slower, $\sqrt{f}$ growth, at around $f \approx \zeta ^2$. This behaviour is clearer for the smaller nonzero choices of $\zeta$ used in Fig. 10. The corrections due to the additional terms in Eq. (27) do not fundamentally change this behaviour for the filter model. The corresponding optimal SNR memory lifetime is

36 $$\begin{aligned} r \tau _{\mathrm {snr}}^{\mathrm {opt}} \approx \frac{a b N}{e [f + (1-f) \zeta ^2]} \, \, \, \, \, \, \text{(at } q^{\mathrm {opt}}\text{) }. \end{aligned}$$

For $\zeta = 0$, $r \tau _{\mathrm {snr}}^{\mathrm {opt}}$ decreases as *f* increases, but for $\zeta = 1$, $r \tau _{\mathrm {snr}}^{\mathrm {opt}}$ is independent of *f*. As *f* increases, the transition from $r \tau _{\mathrm {snr}}^{\mathrm {opt}}$ being independent of *f* to falling as 1/*f* is again sharp, occurring around $f \approx \zeta ^2$. This transition is clear for the filter and serial models in Fig. 10. In the case of the cascade model, however, although $s^{\mathrm {opt}}$ increases with *f*, albeit according to clearly different power laws than for the filter and serial models, the corresponding value of $r \tau _{\mathrm {snr}}^{\mathrm {opt}}$ always decreases as a function of *f*, regardless of $\zeta$.

For the Hebb protocol in Fig. 11, again the small *f* behaviour must be identical to that for the Hopfield protocol in Fig. 10. However, the increase in $\Theta ^{\mathrm {opt}}$ or $s^{\mathrm {opt}}$ with increasing *f* is halted and then reversed as *f* increases further, as the effects of the pairwise synaptic correlations induced by the Hebb protocol are felt. These correlations not only pull down the optimal value of $\Theta ^{\mathrm {opt}}$ or $s^{\mathrm {opt}}$, but they also have a deleterious effect on $r \tau _{\mathrm {snr}}^{\mathrm {opt}}$, changing the 1/*f* behaviour of the filter and serial models in the Hopfield protocol to approximately $1/f^3$ behaviour in the Hebb protocol (obtained by fitting), for $\zeta = 0$. Furthermore, while spontaneous activity can make $r \tau _{\mathrm {snr}}^{\mathrm {opt}}$ independent of *f* before the switch to 1/*f* behaviour in the Hopfield protocol for the filter and serial models, in the Hebb protocol, $r \tau _{\mathrm {snr}}^{\mathrm {opt}}$ always decreases with increasing *f*, for all three complex models considered here.

### Population memory lifetimes

We now turn to population SNR memory lifetimes. Because $\mathrm {SNR}_p(t) \approx \sqrt{g P} \, \mathrm {SNR}(t)$, optimisation of $\tau _{\mathrm {pop}}$ with respect to $\Theta$ or *s* is not affected by the additional factor of $\sqrt{g}$. When we instead optimise population SNR lifetimes with respect to $f = g$, however, the additional factor of $\sqrt{g}$ in $\mu _p(t) / \sigma _p(t)$ compared to $\mu (t) / \sigma (t)$ functionally changes the optima compared to those for a single perceptron, and so we focus on this case. Because of the independence approximation involved in estimating $\tau _{\mathrm {pop}}$ in Sect. 2.4, $\tau _{\mathrm {pop}}$ is only an upper bound on population SNR lifetimes, and this will be implicit below.

For simple, SU synapses, Eq. (20) indicates that $\tau _{\mathrm {snr}}$ and $\tau _{\mathrm {pop}}$ differ in the logarithmic term, with the former having argument $N f^2 p^2/[f + (1-f) \zeta ^2]$ and the latter $\mathscr {N} f^3 p^2/[f + (1-f) \zeta ^2]$. We therefore see immediately that single-perceptron SNR lifetimes with $\zeta = 1$ and population SNR lifetimes with $\zeta = 0$ have identical *f*-dependence. For single-perceptron lifetimes with $\zeta = 0$ and $\zeta = 1$ and population lifetimes with $\zeta = 0$ and $\zeta = 1$, the *f*-dependence under the logarithm is $f^1$, $f^2$, $f^2$ and $f^3$, respectively. The effective power of *f* switches rapidly in the vicinity of $f = \zeta ^2$, in an *N*- or $\mathscr {N}$-dependent way. Because $\mathscr {N} = NP \gg N$, we expect very rapid switching in the population case, and with only very small, even negligible levels of spontaneous activity being required to induce the change in effective power. Above we found for single-perceptron optimal lifetimes that $r \tau _{\mathrm {snr}}^{\mathrm {opt}} \approx p^3 N^2/(4 e)$ at $f^{\mathrm {opt}} = \sqrt{e}/(p^2 N)$ for $\zeta = 0$, and $r \tau _{\mathrm {snr}}^{\mathrm {opt}} \approx p N /(2 e)$ at $f^{\mathrm {opt}} = \sqrt{e} / (p^2 N)^{1/2}$ for $\zeta = 1$. For optimal population lifetimes these become $r \tau _{\mathrm {pop}}^{\mathrm {opt}} \approx p \, \mathscr {N} /(2 e)$ at $f^{\mathrm {opt}} = \sqrt{e} / (p^2 \mathscr {N})^{1/2}$ for $\zeta = 0$, and $r \tau _{\mathrm {pop}}^{\mathrm {opt}} \approx 3 (p \, \mathscr {N}^2)^{1/3} / (4 e)$ at $f^{\mathrm {opt}} = \sqrt{e} / (p^2 \mathscr {N})^{1/3}$. Spontaneous activity changes the *N*-dependence of $r \tau _{\mathrm {snr}}^{\mathrm {opt}}$ from $N^2$ to *N*, and the $\mathscr {N}$-dependence of $r \tau _{\mathrm {pop}}^{\mathrm {opt}}$ from $\mathscr {N}$ to $\mathscr {N}^{2/3}$, which latter is a smaller overall reduction, although in all cases the dependence on *p* involves a positive power. Because the dominant behaviour of SNR lifetimes in the filter and serial models is governed by a similar, single logarithmic term, many of these scaling observations for simple synapses carry over unchanged to these complex synapses.

**Fig. 12:**
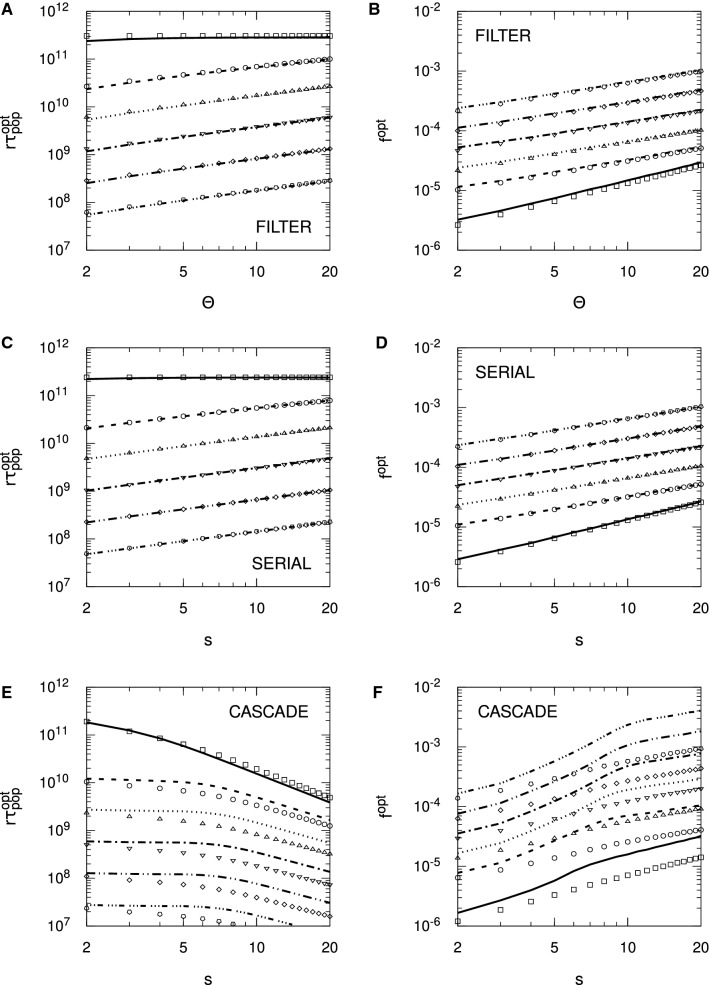
Optimal sparseness in complex synapse models for neuronal populations in the Hopfield protocol. The format of this figure is essentially identical to that in Fig. 8, which shows results for the single-perceptron case. Lines show numerical solutions of the equation $\mu _p(\tau _{\mathrm {pop}}) / \sigma _p(\tau _{\mathrm {pop}}) = 1$ maximised with respect to *f*, so $r \tau _{\mathrm {pop}}^{\mathrm {opt}}$ at $f = f^{\mathrm {opt}}$, while data points show approximate analytical results. We have set $N=10^4$ and $P=10^8$, or $\mathscr {N} = 10^{12}$, in all panels

We examine the behaviour of optimal population SNR lifetimes for complex synapses in Fig. 12. Compared to the single-perceptron optimal SNR lifetimes in Fig. 8, the population results in Fig. 12 are markedly different, particularly for the filter and serial models. For these models, with $\zeta = 0$, $r \tau _{\mathrm {pop}}^{\mathrm {opt}}$ is now approximately independent of $\Theta$ or *s*, while with $\zeta > 0$, $r \tau _{\mathrm {pop}}^{\mathrm {opt}}$ grows as a function of $\Theta$ or *s*. Even with $\zeta = 0.01$, this growth is present and of almost the same profile as that for $\zeta = 1$, while at the single-perceptron level, for smaller choices of *N* it is necessary to take $\zeta$ close to unity to halt the decrease in $r \tau _{\mathrm {pop}}^{\mathrm {opt}}$ with increasing $\Theta$ or *s*. This sensitivity to small, nonzero values of $\zeta$ at the population level is $\mathscr {N}$-dependent, but even with $\mathscr {N} = 10^6$ (e.g. $N = 10^3$ and $P = 10^3$), we only require $\zeta = 0.1$ for $r \tau _{\mathrm {pop}}^{\mathrm {opt}}$ to adopt the same profile as that for $\zeta = 1$. For the cascade model, however, optimal population SNR lifetimes fall with *s* just as they do for single perceptrons. Nonzero $\zeta$ does render $r \tau _{\mathrm {pop}}^{\mathrm {opt}}$ nearly independent of *s* for small *s* ($s \le 6$), but for larger *s*, $r \tau _{\mathrm {pop}}^{\mathrm {opt}}$ falls with *s*. We may quantify the filter and serial models’ population SNR lifetimes as before, using the slowest decaying modes. We obtain

37 $$\begin{aligned} r \tau _{\mathrm {pop}} = \frac{a \, q^2}{f^2} \log _e \frac{\mathscr {N} b f^3}{q^2 [ f + (1-f) \zeta ^2 ]}, \end{aligned}$$

where now we have $f^3$ rather than $f^2$ in the numerator of the logarithm, just as for SU synapses. The optimal values of *f* are now $f^{\mathrm {opt}} = q \sqrt{e} / \sqrt{b \mathscr {N}}$ (cf. $q^2 \sqrt{e} / (b N)$) for $\zeta = 0$ and $f^{\mathrm {opt}} = q^{2/3} \sqrt{e} / (b \mathscr {N})^{1/3}$ (cf. $q \sqrt{e} / \sqrt{b N}$) for $\zeta = 1$. The corresponding optimal memory lifetimes are $r \tau _{\mathrm {pop}}^{\mathrm {opt}} = a b \mathscr {N} / e$ (cf. $a^2 b N^2/(2 e q^2)$) and $r \tau _{\mathrm {pop}}^{\mathrm {opt}} = 3 a (b q \mathscr {N})^{2/3} / (2 e)$ (cf. *abN*/*e*), respectively. The corrections due to the additional terms in the filter models’ results again modify but do not fundamentally alter this behaviour. Thus, at the population level, for $\zeta = 0$, the filter and serial models’ optimal SNR lifetimes are independent of *q*, while at the single perceptron level, they fall as $1/q^2$. However, for $\zeta = 1$, the population lifetimes grow as $q^{2/3}$, while for a single perceptron, they are constant. We cannot obtain similar analytical results for the cascade model, so we fit the numerical results for the cascade model in Fig. 12 to power laws in *s* and $\mathscr {N}$. We find that for larger values of *s*, at the population level $r \tau _{\mathrm {pop}}^{\mathrm {opt}} \sim \mathscr {N}/s^2$ with $f^{\mathrm {opt}} \sim s/\sqrt{\mathscr {N}}$ for $\zeta = 0$ (cf. $\mathscr {N}^2/s^4$ and $s^2/\mathscr {N}$, respectively, for a single perceptron), and $r \tau _{\mathrm {pop}}^{\mathrm {opt}} \sim \mathscr {N}^{2/3}/s^{4/3}$ with $f^{\mathrm {opt}} \sim s^{2/3}/\mathscr {N}^{1/3}$ for $\zeta = 1$ (cf. $\mathscr {N}/s^2$ and $s/\sqrt{\mathscr {N}}$, respectively, for a single perceptron), with the same rapid switching behaviour for intermediate $\zeta$ as for the filter and serial models. The population dynamics soften the fall of $r \tau _{\mathrm {pop}}^{\mathrm {opt}}$ with *s*, but not enough to turn the dependence into growth with *s*.

In Table 2 we summarise the scaling behaviour of $r \tau ^{\mathrm {opt}}$ and $f^{\mathrm {opt}}$ as functions of either *p* or *q* and of either *N* or $\mathscr {N}$, for simple and complex synapses, for both single-perceptron and population results, for $\zeta = 0$ and $\zeta = 1$. In each column, regardless of the model, $f^{\mathrm {opt}}$ scales identically as a function of *q* or $p^{-1}$ and of *N* or $\mathscr {N}$. This is not surprising given that the SU, filter and serial model results for $f^{\mathrm {opt}}$ come from the same dominating logarithmic behaviour, but the cascade results are obtained by fitting numerical matrix data to power laws to extract the behaviour. For $\tau ^{\mathrm {opt}}$, we also obtain the same scaling behaviour as a function of *N* or $\mathscr {N}$ within each column, again regardless of the model. However, the scaling of $\tau ^{\mathrm {opt}}$ with *q* (or $p^{-1}$) within each column does depend on the particular model of plasticity. Across a row, moving from single-perceptron $\zeta =0$ and $\zeta =1$ results to population $\zeta =0$ and $\zeta =1$ results, the dependence of $\tau ^{\mathrm {opt}}$ on *q* (or $p^{-1}$) changes in such a way that increasing *q* (or decreasing *p*) has an increasingly less deleterious effect on memory lifetimes for SU and cascade synapses. For SU synapses, the power of *p* reduces from 3 to 1 to $\frac{1}{3}$, while for cascade synapses the power of *q* (or *s*) changes from $-4$ to $-2$ to $-\frac{4}{3}$. For both SU and cascade synapses, optimal memory lifetimes therefore always decrease as *p* decreases or *s* (the number of metastates) increases, regardless of the level of spontaneous activity, and regardless of whether at a single-perceptron or population level. For filter and serial synapses, however, the power of *q* changes from $-2$ to 0 (i.e. no dependence) to $+\frac{2}{3}$. Increasing the number of serial metastates or filter states available to a filter or serial synapse therefore increases optimal population SNR lifetimes, but only in the presence of spontaneous activity. As Fig. 12 indicates, we need only have very low levels of spontaneous activity to induce this growth of optimal population SNR lifetimes with the number of internal states available to filter or serial synapses.

**Table 2:** Overall dependence of optimal single-perceptron and population SNR memory lifetimes and the corresponding optimal sparseness on model parameters. Here, *q* represents $\Theta$ or *s*, depending on the complex model, and is assumed large

		Single-perceptron		Population	
		$\zeta = 0$	$\zeta = 1$	$\zeta = 0$	$\zeta = 1$
Stochastic updater	$f^{\mathrm {opt}}$	$(p^{-2} / N)^{1}$	$(p^{-2} / N)^{1/2}$	$(p^{-2} / \mathscr {N})^{1/2}$	$(p^{-2} / \mathscr {N})^{1/3}$
	$\tau ^{\mathrm {opt}}$	$p^3 N^2$	*pN*	$p \, {\mathscr {N}}$	$p^{1/3} \mathscr {N}^{2/3}$
Non-cascade	$f^{\mathrm {opt}}$	$(q^2/N)^1$	$(q^2/N)^{1/2}$	$(q^2/\mathscr {N})^{1/2}$	$(q^2/\mathscr {N})^{1/3}$
	$\tau ^{\mathrm {opt}}$	$(N/q)^2$	*N*	$\mathscr {N}$	$(q \, \mathscr {N})^{2/3}$
Cascade	$f^{\mathrm {opt}}$	$(q^2/N)^1$	$(q^2/N)^{1/2}$	$(q^2/\mathscr {N})^{1/2}$	$(q^2/\mathscr {N})^{1/3}$
	$\tau ^{\mathrm {opt}}$	$(N/q^2)^2$	$(N/q^2)^1$	$(\mathscr {N}/q^2)^1$	$(\mathscr {N}/q^2)^{2/3}$

## Discussion

Memory is a complex, multi-level, system-wide phenomenon involving processes occurring over many time scales and across different brain regions, with integrated and orchestrated control processes coordinating, for example, the transition from short- to long-term memory (Eichenbaum and Cohen 2001). Palimpsest models of memory, in which older memories are forgotten as newer ones are stored (Nadal et al. 1986; Parisi 1986), focus on the dynamics of memory storage and retrieval within a single memory system, such as the hippocampal CA3 recurrent network (Andersen et al. 2007). Sparse population coding (see, for example, Csicsvari et al. 2000; Olshausen and Field 2004) enhances memory lifetimes in these memory models by reducing the overall rate of synaptic plasticity at single synapses, so effectively dilating time, and by decorrelating synaptic updates induced by overlapping memories (Tsodyks and Feigel’man 1988). Complex synapse models, involving metaplastic changes in synapses’ internal states without associated changes in synaptic strength, have also been proposed as a way in which to enhance memory lifetimes in palimpsest models (Fusi et al. 2005), whereas we introduced models of integrate-and-express, filter-based synapses as a means of enhancing the stability of developmental patterns of synaptic connectivity in stochastic models of synaptic plasticity (Elliott 2008; Elliott and Lagogiannis 2009).

Understanding the interaction between sparseness and synaptic complexity in palimpsest memory models is therefore crucial (Leibold and Kempter 2008; Rubin and Fusi 2007). Taken at face-value, our results for SNR single-perceptron memory lifetimes support two conclusions. First, optimal single-perceptron SNR lifetimes, optimised with respect to sparseness, require lower synaptic complexity for longer optimal lifetimes. Second, optimal single-perceptron SNR lifetimes, optimised instead with respect to synaptic complexity, again require lower synaptic complexity as sparseness increases for longer optimal lifetimes. These conclusions hold regardless of the level of spontaneous activity, although spontaneous activity can prevent the decrease in optimal single-perceptron memory lifetimes. These conclusions appear to argue in favour of reduced synaptic complexity in real neurons in the presence of sparse population coding, at least at the single-neuron level. However, at the population level, the first of these conclusions is overturned, at least for filter and serial synapses. Critically, even in the presence of low but nonzero levels of spontaneous activity, optimal population SNR lifetimes, optimised with respect to sparseness, increase rather than decrease with synaptic complexity, for filter and serial synapses but not for cascade synapses. At a population level, sparseness, synaptic complexity and, crucially, nonzero spontaneous activity interact to promote increased optimal population SNR memory lifetimes. It is remarkable that non-cascade complex synapse models therefore appear to require the existence of spontaneous activity in a population setting with sparse population coding.

In reaching these conclusions, we have employed two superficially rather different memory storage protocols. First, the Hebb protocol uses two-level inputs $\xi _i \in \{ \zeta , 1 \}$, while the Hopfield protocol uses four-level inputs $\xi _i \in \{ -1 , -\zeta ,$ $+\zeta , +1 \}$, although in stripping out spontaneous activity, the latter reduces to the standard conventions of the Hopfield model with its two-level inputs $\xi _i \in \{ -1 , +1 \}$. However, because we have considered binary strength synapses with $S_i \in \{ -1, +1 \}$, the effective contributions of these two sets of inputs to a perceptron’s activation are identical: in both protocols, $\xi _i S_i$ takes the same four possible values. Second, the Hebb protocol uses the cue and target sub-populations approach to determine the direction of synaptic plasticity, while the Hopfield protocol uses the standard Hopfield rule governed by the product of evoked pre- and postsynaptic activity. However, in both protocols, synapses experience identical potentiating and depressing plasticity induction signals at the same separate rates, *rfg*/2. Furthermore, in both protocols, these induction signals are produced by imposing a pattern of electrical activity on the sub-population of active neurons during memory storage, rather than by allowing neurons’ activities to be generated via direct, afferent synaptic drive. Both protocols therefore implicitly assume executive control of memory storage by other brain regions (see, for example, Eichenbaum and Cohen 2001). These two differences are indeed therefore just superficial, and this is reflected in the fact that the mean activation $\mu (t)$ evolves identically under both protocols. The real difference between the Hebb and Hopfield protocols does not reside in these matters of convention and definition. Rather, it resides in the fact that an active perceptron’s synapses with active inputs experience either only potentiating or only depressing induction signals during memory storage under the Hebb protocol, while in the Hopfield protocol some experience potentiating and others depressing induction signals. This difference gives rise to the Hebb protocol’s complicated equilibrium structure, with its nonzero pairwise and higher-order synaptic correlation functions. Remove this higher-order structure, and the two protocols would have identical statistics for perceptron activation. Indeed, in the limit of small *fgN* in which at most one of a perceptron’s synapses experiences a plasticity induction signal during memory storage, the dynamical difference between the two protocols vanishes and their statistical structures become identical.

Two earlier studies have considered memory lifetimes in complex models of synaptic plasticity in the presence of sparse population coding (Leibold and Kempter 2008; Rubin and Fusi 2007). Leibold and Kempter (2008) used the cue-target protocol that we have adapted and referred to as the Hebb protocol. They employed synaptic strengths $S_i \in \{ 0, 1 \}$ rather than our $S_i \in \{ -1, +1 \}$, although this difference is unimportant because it just amounts to an effective re-definition of the firing threshold $\vartheta$ (Elliott and Lagogiannis 2012). They also employed two-level activities, but with $\xi _i \in \{ 0, 1 \}$, so without considering the possible influence of nonzero spontaneous activity, $\zeta > 0$, on memory lifetimes. Rubin and Fusi (2007) used the Hopfield protocol with two-level activities, $\xi _i \in \{ -1, +1 \}$, interpreting $\xi _i = -1$ as spontaneous activity and $\xi _i = +1$ as evoked activity, and stressed the importance of considering the impact of spontaneous activity on memory lifetimes. We have modelled spontaneous activity in the Hopfield protocol by moving to four-level inputs, but as indicated, this approach is essentially equivalent to two-level inputs for synapses with $S_i \in \{ -1 , +1 \}$ in terms of the overall statistical structure of perceptron activation. However, by using four activity levels, we are able to consider varying $\zeta$ over its allowed range in order to explore the impact of different degrees of spontaneous activity on memory lifetimes. A significant difference between our approach and that of Rubin and Fusi (2007) is that we do not allow spontaneous activity to induce synaptic plasticity, a position that we consider to be mandated by a broadly BCM (Bienenstock et al. 1982) view of synaptic plasticity, as discussed earlier. Finally, our respective definitions of the memory signal, from which SNR memory lifetimes are obtained, differ in a population setting. Rubin and Fusi (2007) define this signal over the entire population of neurons, while we define it over only that sub-population of neurons that are directly involved in memory storage (or the equivalent of Leibold and Kempter (2008)’s target sub-population). This difference leads to different scaling behaviours of optimal population SNR memory lifetimes as a function of the sparseness of the population coding.

The difference between the scaling behaviours of optimal SNR memory lifetimes (optimised with respect to sparseness) in the single-perceptron and population cases is intriguing. Furthermore, the role of even very small levels of spontaneous activity in enhancing optimal population SNR lifetimes with increasing synaptic complexity in non-cascade models is fascinating. However, we have cautioned against over-interpreting results from an SNR analysis of memory lifetimes. This analysis depends on the distribution of $h_0$ being tightly concentrated around its supra-threshold mean. We have shown in earlier work that this requirement is often not satisfied, and that a FPT approach is required to examine memory lifetimes away from this regime (Elliott 2016a, 2017a, 2020). Here, for simple synapses, we have explicitly seen that the single-perceptron SNR analysis breaks down in the limit of small *f*, and so it cannot probe the very sparse coding regime. The explanation for this failure is straightforward: as *f* is reduced, the initial SNR $\mu (0) / \sigma (0)$ reduces, and below some threshold value of *f* the SNR validity condition $\mu (0) / \sigma (0) \gtrapprox 2$ fails. For a single perceptron, we saw that this condition is $N f^2 p^2 / \left[ f + (1-f) \zeta ^2 \right] \gtrapprox 4$ (for either protocol). Plugging in $f^{\mathrm {opt}}$ for SU synapses with $\zeta = 0$ and $\zeta = 1$, this condition becomes $\sqrt{e} \gtrapprox 4$ and $e \gtrapprox 4$, respectively, where we saw the former case earlier. Both conditions are violated, although with spontaneous activity the violation is not so great. Although we have not extended our FPT analysis of filter-based synapses (Elliott 2017a, 2020) to the sparse coding regime considered here, the same issues arise with complex synapses. Therefore, we fully expect single-perceptron SNR optimality conditions to be violated for complex synapses, too.

Whether population SNR optimality conditions are violated, in either simple or complex models, is unclear. We would need to extend our single-perceptron FPT analysis to a population setting. Furthermore, this extended analysis would need to be reducible to the population SNR analysis with its rather coarse approximation that neurons’ activities evolve independently, despite synaptic coupling. However, it is extremely tempting to speculate that the simple synapse condition for population SNR validity is just the obvious generalisation, namely $\mu _p(0) / \sigma _p(0) \gtrapprox 2$. Using the population results for $f^{\mathrm {opt}}$ for simple synapses, this condition becomes the false $e \gtrapprox 4$ for $\zeta = 0$ and the true $e^{3/2} \gtrapprox 4$ for $\zeta = 1$. It is thus quite remarkable that if this speculation is borne out by a more careful analysis, then optimal population SNR memory lifetimes for simple synapses are valid in the presence of spontaneous activity.

## Figures and Tables

**Fig. 13:**
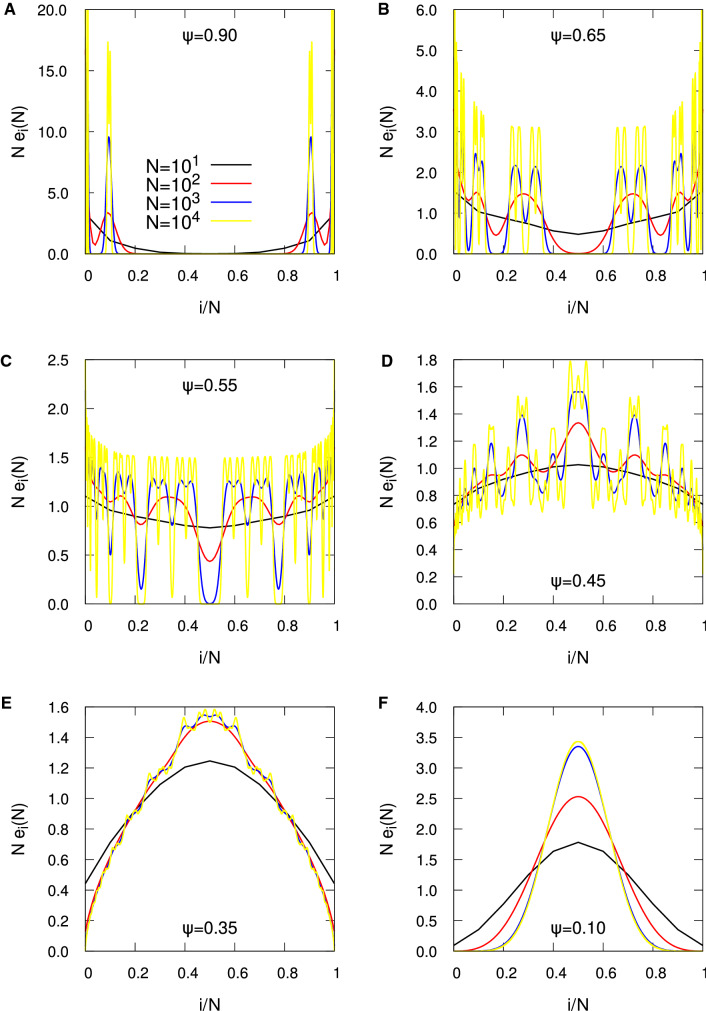
Hebb equilibrium probability distribution for stochastic updater synapses. Each panel shows $e_i(N)$ plotted against *i* for a fixed choice of $\psi = f p$ as indicated, and for different choices of *N*, with both $e_i(N)$ and *i* scaled appropriately to permit comparison

## Data Availability

Available upon request.

## Transition matrix elements and jump moments

Table 3 provides an additional table of frequently used mathematical symbols and their meanings used exclusively in the appendices.

To obtain FPT lifetimes for SU synapses, we require $\mathsf {Prob}[ \, h \, | \, h' \, ]$, or the induced jump moments. For synaptic strengths $\pm 1$, the number of synapses of strength $+1$ uniquely determines *h*(*t*). Let $N_{\mathrm {eff}}$ of a perceptron’s synapses be active during the storage of $\underline{\xi }^0$. Consider first the Hebb protocol. Immediately before the storage of any subsequent non-tracked memory, let *i* of these $N_{\mathrm {eff}}$ synapses have strength $+1$ and *j* of the other $N - N_{\mathrm {eff}}$ synapses have strength $+1$. Then,

38 $$\begin{aligned} N h' = (2i - N_{\mathrm {eff}}) + \zeta \left[ 2j - (N - N_{\mathrm {eff}}) \right] . \end{aligned}$$

Similarly, immediately after the storage of this non-tracked memory, let *k* and *l* synapses out of the $N_{\mathrm {eff}}$ and $N - N_{\mathrm {eff}}$ synapses, respectively, have strength $+1$, so that

39 $$\begin{aligned} N h = (2k - N_{\mathrm {eff}}) + \zeta \left[ 2l - (N - N_{\mathrm {eff}}) \right] . \end{aligned}$$

Then, the transition operator $\mathbb {T}_N = (1 - g) \, \mathbb {I} \otimes \cdots \otimes \mathbb {I} + {\textstyle \frac{1}{2}}g \, \mathbb {K}_+ \otimes \cdots \otimes \mathbb {K}_+ + {\textstyle \frac{1}{2}}g \, \mathbb {K}_- \otimes \cdots \otimes \mathbb {K}_-$ induces the corresponding transition probability

40 $$\begin{aligned} \mathsf {Prob}[ \, k,l \, | \, i,j \, ]&= (1 - g) \, \delta _{k,i} \, \delta _{l,j} \nonumber \\&\quad + {\textstyle \frac{1}{2}}\, g \, \Phi _{k - i}^{N_{\mathrm {eff}}- i} (\psi ) \, \Phi _{l-j}^{N - N_{\mathrm {eff}}- j} (\psi ) \nonumber \\&\quad + {\textstyle \frac{1}{2}}\, g \, \Phi _{i-k}^i(\psi ) \, \Phi _{j-l}^j(\psi ), \end{aligned}$$

**Table 3:** List of main mathematical symbols and their meanings appearing in the appendices

Symbol	Meaning
$\Phi _m^n(p)$	$\Phi _m^n(p) = {^n C_m} p^m (1-p)^{n-m}$, the binomial distribution’s mass function
$\underline{e}(N)$	Normalised unit eigenvector of $(N+1) \times (N+1)$ matrix with transition elements given in Eq. (42)
$F_N(z)$	$F_N(z) = \sum _{i=0}^N e_i(N) z^i$, probability generation function of the components of $\underline{e}(N)$
*G*(*z*, *w*)	$G(z,w) = \sum _{N=0}^\infty F_N(z) w^N$, ordinary generating function for the PGFs $F_N(z)$
$\underline{\varepsilon }(N)$, $\mathscr {F}_N(z)$, $\mathscr {G}(z,w)$	Equivalents of $\underline{e}(N)$, $F_N(z)$, *G*(*z*, *w*) for model-independent arguments
*H*(*x*)	*G*(*z*, *w*) re-expressed in terms of the single dependent variable $x = w (1-z) / [ 2 - w (1+z) ]$
$\kappa _n$	Coefficient in power series for *H*(*x*), or *n* synapses’ equilibrium strength correlation coefficient
*K*(*z*)	Exponential generating function for the correlation coefficients $\kappa _n$
$\psi _j$	$\psi _j = \psi \left( 1 - \psi \right) ^j$

where $\delta _{i,j}$ is the Kronecker delta function, and $\Phi _m^n(p) = {^n C_m} \, p^m \, (1-p)^{n-m}$ is the binomial probability distribution with ${^n C_m}$ being the binomial coefficient. The three terms correspond to the three parts of $\mathbb {T}_N$, with the last two just being products of the probabilities for the possible ways in which the different sets of synapses can change strength to give the required transition process. We can also obtain a similar result for the Hopfield protocol. In this case, let *i* of the $N_{\mathrm {eff}}$ synapses and *j* of the other $N - N_{\mathrm {eff}}$ synapses contribute positively to $h'$; and similarly *k* and *l*, respectively, to *h*. For example, a synapse with a component $\xi ^0 = +1$ (or $+\zeta$) and $S(t) = +1$ and a synapse with a component $\xi ^0 = -1$ (or $-\zeta$) and $S(t) = -1$ both contribute positively to *i* (or *j*). We again just have $N h' = (2i - N_{\mathrm {eff}}) + \zeta \left[ 2j - (N - N_{\mathrm {eff}}) \right]$, and similarly for *Nh*. Then, from $\mathbb {T}_N = (1-g) \, \mathbb {I} \otimes \cdots \otimes \mathbb {I} + g \, \mathbb {K} \otimes \cdots \otimes \mathbb {K}$, we obtain

41 $$\begin{aligned} \mathsf {Prob}[ \, k,l \, | \, i,j \, ]&= (1 - g) \, \delta _{k,i} \, \delta _{l,j} \nonumber \\&\quad + \, g \, \sum _{m=0}^i \Phi _m^i \big ( {\textstyle \frac{1}{2}}\psi \big ) \, \Phi _{k+m-i}^{N_{\mathrm {eff}}- i} \big ( {\textstyle \frac{1}{2}}\psi \big ) \nonumber \\&\quad \times \sum _{n=0}^j \Phi _m^j \big ( {\textstyle \frac{1}{2}}\psi \big ) \, \Phi _{l+n-j}^{N - N_{\mathrm {eff}}- j} \big ( {\textstyle \frac{1}{2}}\psi \big ), \end{aligned}$$

where the first sum arises because $m \in \{0, \ldots , i\}$ out of the *i* synapses can change strength and then $k+m-i$ of the $N_{\mathrm {eff}}- i$ synapses must also change strength for the $i \rightarrow k$ transition, and similarly for the second sum. These probabilities $\mathsf {Prob}[ \, k,l \, | \, i,j \, ]$ uniquely determine $\mathsf {Prob}[ \, h \, | \, h' \,]$, from which we can obtain the jump moments in Eq. (5). The final results are given in Eqs. (21) and (22). The second jump moment depends explicitly on $N_{\mathrm {eff}}$, so we denote it as $B(h' \, | \, N_{\mathrm {eff}})$. It is convenient to write $B(h' \, | \, N_{\mathrm {eff}}) = \psi g \, B_0 ( N_{\mathrm {eff}}) + \psi ^2 g \, ( h' )^2$, so that $B_0( N_{\mathrm {eff}})$ is, up to a multiplicative factor, the $h'$-independent part of $B(h' \, | \, N_{\mathrm {eff}})$.

To obtain FPT lifetimes from the FPE approach, we must solve Eq. (6) with its $N_{\mathrm {eff}}$-dependent second jump moment for any particular choice of $N_{\mathrm {eff}}$. For this given value, we must average the resulting solution $\tau _{\mathrm {mfpt}}(h_0 \, | \, N_{\mathrm {eff}})$ over the initial distribution of $h_0$, which also depends on $N_{\mathrm {eff}}$, and then average over $N_{\mathrm {eff}}$ according to its binomial distribution, obtaining $\tau _{\mathrm {mfpt}} = \langle \langle \tau _{\mathrm {mfpt}}(h_0 \, | \, N_{\mathrm {eff}}) \rangle _{h_0 > \vartheta } \rangle _{N_{\mathrm {eff}}}$. However, for large enough *fN*, it is sufficient to average $B_0(N_{\mathrm {eff}})$ over the distribution of $N_{\mathrm {eff}}$ and just use the average jump moment $\psi g \, \langle B_0(N_{\mathrm {eff}}) \rangle _{N_{\mathrm {eff}}}$ in Eq. (6). This “pre-averaging” method is similar to a mean field approximation, but goes beyond just replacing $N_{\mathrm {eff}}$ with its mean value, *fN*. We then average the resulting solution $\tau _{\mathrm {mfpt}}(h_0)$ over $h_0$, where the unconditional statistics of $h_0 = h(0)$ are given in Eqs. (7) and (11) with the correlations in Eq. (19). Because the FPE is valid only to second order, it suffices to take the distribution of $h_0$ as a Gaussian with these first- and second-order statistics.

Although $\mathsf {Prob}[ k,l | i,j ]$ determines $\mathsf {Prob}[ h | h' ]$ uniquely, the reverse is in general not the case in the presence of nonzero spontaneous activity. In particular, the equation $N h' = (2i - N_{\mathrm {eff}}) + \zeta \left[ 2j - (N - N_{\mathrm {eff}}) \right]$ may have multiple solutions for *i* and *j*, given a value of $h'$, depending on the value of $\zeta$. To avoid this awkwardness, for determining FPT lifetimes according to the exact, MIE approach of Eq. (4) or its continuum limit, we restrict to the specific case of $\zeta = 0$. Then, the contributions to *h*(*t*) from the inactive inputs drop out, and we need only work with $\mathsf {Prob}[ \, k \, | \, i \, ]$, with the transition processes involving *j* and *l* being irrelevant. Since $N h' = (2i - N_{\mathrm {eff}})$ and $N h = (2k - N_{\mathrm {eff}})$, $\mathsf {Prob}[ \, k \, | \, i \, ]$ uniquely determines $\mathsf {Prob}[ \, h \, | \, h' \,]$ and *vice versa*. As with Eq. (6), Eq. (4) is then conditioned on $N_{\mathrm {eff}}$ synapses contributing positively to the perceptron’s activation during the storage of $\underline{\xi }^0$, and so we must also solve Eq. (4) for each value of $N_{\mathrm {eff}}$ and compute the same double average, $\tau _{\mathrm {mfpt}} = \langle \langle \tau _{\mathrm {mfpt}}(h_0 \, | \, N_{\mathrm {eff}}) \rangle _{h_0 > \vartheta } \rangle _{N_{\mathrm {eff}}}$. Where feasible, we always perform this exact calculation. For larger choices of *N* and values of *f* closer to unity (for which *fN* remains sizeable), we move to the mean field approximation, setting $N_{\mathrm {eff}}= f N$ (or its closest integer), which is an excellent approximation that makes the calculations tractable. For even larger *N* ($N = 10^6$), we move to the integral equation form of Eq. (4), corresponding to a continuum limit for *h*. This limit also works well for smaller values of *N*, but we prefer exact methods where possible. Unlike the FPE approach, we need the exact distribution of $h_0$ in the MIE approach, or a good approximation to it, to average correctly over $h_0$, and so we need the equilibrium distribution of all synapses’ strengths. We give the details of the calculation for the Hebb protocol in Appendix B.

## Hebb equilibrium structure for simple synapses

We require the unit eigenstate of the operator ${\textstyle \frac{1}{2}}\, \mathbb {K}_+ \otimes \cdots \otimes \mathbb {K}_+ + {\textstyle \frac{1}{2}}\, \mathbb {K}_- \otimes \cdots \otimes \mathbb {K}_-$ for all *N* synapses, where $\mathbb {K}_\pm$ are given in Eq. (16). This operator induces the transition probabilities

42 $$\begin{aligned} \mathsf {Prob}[ \, k \, | \, i \, ] = {\textstyle \frac{1}{2}}\Phi _{k-i}^{N-i}(\psi ) + {\textstyle \frac{1}{2}}\Phi _{i-k}^i (\psi ), \end{aligned}$$

for the number of synapses of strength $+1$ in equilibrium (cf. Eq. (40)). These probabilities are the elements of an $(N+1) \times (N+1)$ matrix whose unit eigenvector determines the equilibrium distribution. Let the $(N+1)$-dimensional vector $\underline{e}(N)$ with components $e_i(N)$, $i=0, \ldots , N$, be this eigenvector, with $\sum _{i=0}^N e_i(N) = 1$. Then $e_i(N)$ is the probability that *i* synapses have strength $+1$ in equilibrium, where we indicate the dependence of $e_i(N)$ on *N* for clarity. We define the probability generating function (PGF) $\mathscr {G}_i(z) = \sum _{i=0}^N z^k \, \mathsf {Prob}[ \, k \, | \, i \, ]$ for the columns of the transition matrix, obtaining

43 $$\begin{aligned} \mathscr {G}_i(z) = {\textstyle \frac{1}{2}}z^i \left[ (1 - \psi ) + \psi \, z \right] ^{N-i} + {\textstyle \frac{1}{2}}\left[ \psi + (1 - \psi ) z \right] ^i,\nonumber \\ \end{aligned}$$

and also define the PGF for the components of its unit eigenvector $\underline{e}(N)$ by writing $F_N(z) = \sum _{i=0}^N e_i(N) \, z^i$. Then, the eigenvalue equation can be written as:

44 $$\begin{aligned} F_N(z)&= {\textstyle \frac{1}{2}}\left[ (1 - \psi ) + \psi \, z \right] ^N F_N \left( \frac{z}{(1 - \psi ) + \psi \, z} \right) \nonumber \\&\quad + {\textstyle \frac{1}{2}}\, F_N \, \left( \psi + (1 - \psi ) z \right) . \end{aligned}$$

Forming an ordinary generating function (OGF) by writing $G(z,w) = \sum _{N=0}^\infty F_N(z) \, w^N$, we obtain

45 $$\begin{aligned} G(z,w)&= {\textstyle \frac{1}{2}}\, G \left( \frac{z}{(1 - \psi ) + \psi \, z} , \left[ (1 - \psi ) + \psi \, z \right] w \right) \nonumber \\&\quad + {\textstyle \frac{1}{2}}\, G \left( \psi + (1 - \psi ) z , w \right) . \end{aligned}$$

This equation must be solved subject to the two boundary conditions $G(1,w) = 1/(1-w)$ and $G(z,0) = F_0(z) \equiv 1$.

Although Eq. (45) is a nasty functional equation, we can exploit very general, model-independent arguments to simplify it. For indistinguishable synapses, marginalising a general equilibrium distribution $\underline{\mathscr {A}}_{N+1}$ over one synapse must give $\underline{\mathscr {A}}_N$. Let $\varepsilon _i(N)$ be the probability that any *i* out of *N* synapses have strength $+1$ in this general equilibrium distribution $\underline{\mathscr {A}}_N$. The probability of any particular configuration of *i* such synapses having strength $+1$ is then just $\varepsilon _i(N) / {^N}C_i$. Considering an additional synapse added to these *N* synapses, it could have strength $-1$ or $+1$, and these two new, particular configurations have probabilities $\varepsilon _i(N+1) / {^{N+1}} C_i$ and $\varepsilon _{i+1} (N+1) / {^{N+1}} C_{i+1}$, respectively. So, we must have

46 $$\begin{aligned} \frac{1}{{^N} C_i} \, \varepsilon _i(N) = \frac{1}{{^{N+1}} C_i} \, \varepsilon _i(N+1) + \frac{1}{{^{N+1}} C_{i+1}} \, \varepsilon _{i+1} (N+1),\nonumber \\ \end{aligned}$$

which represents the result of marginalising $\underline{\mathscr {A}}_{N+1}$ over one synapse’s strength to obtain $\underline{\mathscr {A}}_N$. Then,

47 $$\begin{aligned} \varepsilon _i(N) = \frac{N+1-i}{N+1} \, \varepsilon _i(N+1) + \frac{i+1}{N+1} \, \varepsilon _{i+1} (N+1).\nonumber \\ \end{aligned}$$

This equation has natural boundary conditions: putting $i=N+1$ or $i=-1$ gives zero on the RHS, respecting the convention that $\varepsilon _i(N) = 0$ for $i<0$ and $i>N$. Writing the PGF $\mathscr {F}_N(z) = \sum _{i=0}^N \varepsilon _i(N) \, z^i$ and using these boundary conditions, the PGF must satisfy the ordinary differential equation

48 $$\begin{aligned} (1-z) \, \frac{d \mathscr {F}_{N+1} (z)}{dz} + (N+1) \mathscr {F}_{N+1} (z) = (N+1) \mathscr {F}_N (z).\nonumber \\ \end{aligned}$$

Then writing the OGF $\mathscr {G}(z,w) = \sum _{N=0}^\infty \mathscr {F}_N(z) \, w^N$, we obtain the partial differential equation

49 $$\begin{aligned} (1-z) \, \frac{\partial \mathscr {G}(z,w)}{\partial z} + w (1-w) \, \frac{\partial \mathscr {G}(z,w)}{\partial w} = w \, \mathscr {G} (z,w),\nonumber \\ \end{aligned}$$

which is subject to the boundary conditions $\mathscr {G}(1,w) = 1/(1-w)$ and $\mathscr {G}(z,0) = 1$. The general solution of this equation can be written in the form:

50 $$\begin{aligned} \mathscr {G} (z,w) = \frac{1}{w(1-z)} \, \mathscr {H} \left( \frac{w(1-z)}{2 - w (1+z)} \right) \end{aligned}$$

for an arbitrary (at least once-differentiable) function $\mathscr {H}(x)$, where the two boundary conditions at $z=1$ and $w=0$ impose the same requirement, that $\mathscr {H}(x) / x \rightarrow 2$ as $x \rightarrow 0$. The solution in Eq. (50) imposes a functional constraint on the form of $\mathscr {G} (z,w)$ in any model with indistinguishable synapses.

Applying the general form in Eq. (50) to the particular case in Eq. (45), so writing *G*(*z*, *w*) in terms of some function *H*(*x*) where $x = w(1-z)/[2 - w(1+z)]$, we reduce the functional equation involving a function in two variables to the much simpler and more symmetric functional equation

51 $$\begin{aligned} H(x) = \frac{1}{2(1 - \psi )} \left[ H \left( \frac{(1 - \psi ) x}{1 + \psi x} \right) + H \left( \frac{(1 - \psi ) x}{1 - \psi x} \right) \right] ,\nonumber \\ \end{aligned}$$

in just one variable. We solve this equation using a power series solution. There are no even terms (because $H(0) \equiv 0$ guarantees that *H*(*x*)/*x* is finite as $x \rightarrow 0$), so we write

52 $$\begin{aligned} H(x) = 2 x \sum _{i=0}^\infty \kappa _{2i} \, x^{2i}, \end{aligned}$$

with $\kappa _0 \equiv 1$ satisfying the boundary conditions. The coefficients $\kappa _{2i}$ must then satisfy the infinite tower of recurrence relations

53 $$\begin{aligned} \frac{\kappa _{2i}}{(2i)!} = \sum _{j=0}^i \frac{\kappa _{2j}}{(2j)!} (1 - \psi )^{2j} \frac{\psi ^{2(i-j)}}{[2(i-j)]!}. \end{aligned}$$

These equations can be solved iteratively to any desired order.

Given the $\kappa _{2i}$ coefficients, we then have *H*(*x*), with *G*(*z*, *w*) following directly as:

54 $$\begin{aligned} G(z,w) = \sum _{i=0}^\infty \kappa _{2i} \, \frac{w^{2i} (\frac{1-z}{2})^{2i}}{\left[ 1 - w \, \left( \frac{1+z}{2} \right) \right] ^{2i+1}}. \end{aligned}$$

Then, $F_N(z)$ follows, which can be written in the form:

55 $$\begin{aligned} F_N(z) = \sum _{i=0}^N {^N}C_i \, \kappa _i \, \left( \frac{1-z}{2} \right) ^i \, \left( \frac{1+z}{2} \right) ^{N-i}, \end{aligned}$$

where we define the odd coefficients $\kappa _{2i+1} \equiv 0$ to make the expression for $F_N(z)$ take its simplest form. By definition $F_N(z) \equiv \sum _{i=0}^N e_i(N) \, z^i$, so by reading off the coefficient of $z^i$ in Eq. (55) we obtain $e_i(N)$ in terms of the $\kappa _{2j}$ coefficients. From the definition of $F_N(z)$ as the PGF for the number of synapses with strength $+1$ in equilibrium, the equilibrium distribution $\underline{A}_N$ takes the form:

56 $$\begin{aligned} \underline{A}_N = \sum _{i=0}^N \frac{e_i(N)}{{^N}C_i} \, \sum _\mathscr {P} \Bigg [ \underbrace{\begin{pmatrix} 1 \\ 0 \end{pmatrix} \otimes \cdots \otimes \begin{pmatrix} 1 \\ 0 \end{pmatrix} }_{N-i} \otimes \underbrace{\begin{pmatrix} 0 \\ 1 \end{pmatrix} \otimes \cdots \otimes \begin{pmatrix} 0 \\ 1 \end{pmatrix} }_i \Bigg ],\nonumber \\ \end{aligned}$$

where $\sum _\mathscr {P}$ denotes a sum over all ${^N}C_i$ combinations of the *N* indicated tensor products involving *i* synapses of strength $+1$ and $N-i$ of strength $-1$, and the $e_i(N)$ can be expressed in terms of the $\kappa _{2j}$ via Eq. (55).

Although this completely solves the problem of finding the Hebb equilibrium distribution, in fact we can write $\underline{A}_N$ directly in terms of the $\kappa _i$ coefficients. To see this, we first evaluate $F_N(z)$ at $z = -1$ using Eq. (55), giving $F_N(-1) \equiv \kappa _N$. But $F_N(-1) = \sum _{i=0}^N (-1)^i \, e_i(N)$, and this alternating sum is, up to an overall sign, just the equilibrium correlation coefficient $\mathsf {E}[ S_1(\infty ) \times \cdots \times S_N(\infty )]$, since

57 $$\begin{aligned}&\mathsf {E}[ S_1(\infty ) \times \cdots \times S_N(\infty ) ] \nonumber \\&\quad = (-1)^N \, \times \, e_0(N) + (-1)^{N-1} \, \times \, e_1(N) \nonumber \\&\qquad + \cdots + (-1)^0 \, \times \, e_N(N) \nonumber \\&\quad = (-1)^N \sum _{i=0}^N (-1)^i \, e_i(N). \end{aligned}$$

So $\mathsf {E}[ S_1(\infty ) \times \cdots \times S_N(\infty ) ] = (-1)^N F_N(-1) \equiv (-1)^N \kappa _N$. But $\kappa _{2i+1} \equiv 0$, so we can just drop the parity factor $(-1)^N$. Hence, $\kappa _N$ is the equilibrium correlation function between the strengths of *N* synapses, for any choice of *N*. We must therefore be able to expand $\underline{A}_N$ directly in terms of these correlation functions. Equation (56) writes $\underline{A}_N$ in terms of the two orthogonal vectors $(1,0)^{\mathrm {T}}$ and $(0,1)^{\mathrm {T}}$. Although these definite strength states are the natural ones, we can instead expand the equilibrium state using a different pair of orthogonal vectors. In particular, we may use the pair $\underline{A}_1 = \frac{1}{2}(+1,+1)^{\mathrm {T}}$ and $\underline{A}^\perp _1 = \frac{1}{2}(+1,-1)^{\mathrm {T}}$, where the former is just the monosynaptic equilibrium distribution. Then, we may instead write

58 $$\begin{aligned} \underline{A}_N = \sum _{i=0}^N \kappa _i \sum _\mathscr {P} \big [ \underbrace{\underline{A}_1 \otimes \cdots \otimes \underline{A}_1}_{N-i} \otimes \underbrace{\underline{A}^\perp _1 \otimes \cdots \otimes \underline{A}^\perp _1}_i \big ]. \end{aligned}$$

Because $\kappa _{2i+1} = 0$, only an even number of $\underline{A}^\perp _1$ vectors can appear in each term. We may confirm by explicit calculation that Eqs. (56) and (58) are equivalent representations of the same equilibrium state $\underline{A}_N$, and we may also confirm that the form in Eq. (58) has the correct marginal and correlational structure. For example, to compute a correlation function involving *j* out of the *N* synapses, we need to marginalise over $N-j$ synapses. This marginalisation is achieved just by summing over their states, which we do by dotting through with the 2-dimensional vector $\underline{1}$ in the $N-j$ relevant places in the tensor product. To obtain expectation values of the other *j* synapses’ strengths, we just dot through with $\underline{\Omega }$ in the *j* relevant places. But, $\underline{1} \equiv 2 \underline{A}_1$ and $\underline{\Omega } \equiv - 2 \underline{A}^\perp _1$, so Eq. (58) is just a disguised expansion in the orthogonal vectors $\underline{1}$ and $\underline{\Omega }$ that must be used when computing the equilibrium correlation functions. Equation (58) is therefore the only possible form with the requisite correlational structure.

Although in obtaining Eq. (53) we have essentially found the Hebb equilibrium distribution, using it to obtain the $\kappa _{2i}$ is awkward. For example, the first few $\kappa _{2i}$ are

59 $$\begin{aligned} \left. \begin{array}{ccl} \kappa _0 &{} = &{} 1, \\ \kappa _2 &{} = &{} \frac{\psi }{2-\psi }, \\ \kappa _4 &{} = &{} \frac{\psi ^2}{(2-\psi )^2} \frac{( 6 - 10 \psi + 5 \psi ^2 )}{(2 - 2 \psi + \psi ^2)}, \\ \kappa _6 &{} = &{} \frac{\psi ^3}{(2-\psi )^3} \frac{( 90 - 450 \psi + 1013 \psi ^2 - 1276 \psi ^3 + 929 \psi ^4 - 366 \psi ^5 + 61 \psi ^6 )}{(6 - 18 \psi + 29 \psi ^2 - 28 \psi ^3 + 17 \psi ^4 - 6 \psi ^5 + \psi ^6)} , \end{array} \right\} \end{aligned}$$

and they become increasingly complicated as *i* increases. We can compute these coefficients numerically for any given value of $\psi$, but high precision is required to obtain stable results, with more precision required as *N* increases. Rather than using Eq. (53) directly, we instead define the exponential generating function (EGF) of the coefficients $\kappa _{2i}$,

60 $$\begin{aligned} K(z) = \sum _{i=0}^\infty \frac{\kappa _{2i}}{(2i)!} \, z^{2i}. \end{aligned}$$

This EGF undoes the convolution structure in Eq. (53), and we obtain the *q*-like equation (see, for example, Andrews et al. 1999)

61 $$\begin{aligned} K(z) = K \big ( (1 - \psi ) z \big ) \cosh \psi z. \end{aligned}$$

The solution follows by iteration, so that

62 $$\begin{aligned} K(z) = \prod _{j=0}^\infty \cosh \psi (1 - \psi )^j z, \end{aligned}$$

where we have used $K(0) \equiv 1$. This EGF can be evaluated in closed form for the three cases of $\psi = 1$, $\psi = {\textstyle \frac{1}{2}}$ and $\psi = 0$ (or strictly in the limit $\psi \rightarrow 0$), giving

63 $$\begin{aligned} K(z) = \left\{ \begin{matrix} 1 &{}\quad \text{ for } \psi = 0 \\ {\displaystyle \frac{\sinh z}{z}} &{} \quad \text{ for } \psi = {\textstyle \frac{1}{2}}\\ \cosh z &{}\quad \text{ for } \psi = 1 \end{matrix} \right. . \end{aligned}$$

The coefficients $\kappa _{2i}$ follow as: $\kappa _{2i} = \delta _{i,0}$ for $\psi = 0$; $\kappa _{2i} = 1/(1+2i)$ for $\psi = {\textstyle \frac{1}{2}}$; and $\kappa _{2i} = 1$ for $\psi = 1$. Plugging these into Eq. (55), we obtain

64 $$\begin{aligned} F_N(z) = \left\{ \begin{matrix} {\displaystyle \left( \frac{1+z}{2} \right) ^N} &{} \quad \text{ for } \psi = 0 \\ {\displaystyle \frac{1}{N+1} \frac{1 - z^{N+1}}{1-z}} &{}\quad \text{ for } \psi = {\textstyle \frac{1}{2}}\\ {\displaystyle {\textstyle \frac{1}{2}}\left( 1 + z^N \right) } &{} \quad \text{ for } \psi = 1 \end{matrix} \right. . \end{aligned}$$

Thus, for $\psi = 0$, $e_i(N) = {^N}C_i/2^N$, so binomially distributed with probability ${\textstyle \frac{1}{2}}$; for $\psi = {\textstyle \frac{1}{2}}$, $e_i(N) = 1/(N+1)$, so uniformly distributed; for $\psi = 1$, $e_i(N) = {\textstyle \frac{1}{2}}\left( \delta _{i,0} + \delta _{i,N} \right)$, so bimodally distributed with equiprobable spikes at $i=0$ and $i=N$ only. The case of $\psi = 0$ (or $\psi \rightarrow 0$) corresponds to the distribution $\underline{A}_N = \underline{A}_1 \otimes \cdots \otimes \underline{A}_1$.

Away from these exact cases, we approximate *K*(*z*) by considering only a finite number of terms in the product for the EGF in Eq. (62),

65 $$\begin{aligned} K_m(z) = \prod _{j=0}^m \cosh \psi (1 - \psi )^j z. \end{aligned}$$

Writing $\cosh$ in its exponential form and considering all combinations of products of the individual terms, the coefficient of $z^i$ in $K_m(z)$ is

66 $$\begin{aligned} \left[ z^i \right] K_m(z)&= \frac{\left[ 1 + (-1)^i \right] }{2} \frac{1}{i!} \frac{1}{2^m} \nonumber \\&\quad \times \sum _{\pm \cdots \pm } \left( \psi _0 \pm \psi _1 \pm \psi _2 \pm \cdots \pm \psi _m \right) ^i, \end{aligned}$$

where we write $\psi _j = \psi (1 - \psi )^j$, and the sum is over all $2^m$ possible combinations of signs. For $m=0$, the sum just means $\psi _0$. Thus, we write

67 $$\begin{aligned} \kappa _i^{(m)} {=} \frac{\left[ 1 + (-1)^i \right] }{2} \frac{1}{2^m} \sum _{\pm \cdots \pm } \left( \psi _0 {\pm } \psi _1 \pm \psi _2 \pm \cdots \pm \psi _m \right) ^i,\nonumber \\ \end{aligned}$$

and then $\lim _{m \rightarrow \infty } \kappa _i^{(m)} = \kappa _i$. For $\psi > 0$, $\psi _j$ falls to zero geometrically fast as *j* increases, so the convergence is rapid. Thus, a controlled approximation replaces the coefficients $\kappa _i$ with their truncated forms $\kappa _i^{(m)}$, where we only need to take *m* large enough for good convergence. For notational simplicity, we write Eq. (67) in the form:

68 $$\begin{aligned} \kappa _i^{(m)} = \frac{\left[ 1 + (-1)^i \right] }{2} \frac{1}{2^m} \sum _\alpha \, p_\alpha ^i, \end{aligned}$$

where the sum over $p_\alpha ^i$ is shorthand for the full sum in Eq. (67). Inserting this into Eq. (55), we obtain the PGF

69 $$\begin{aligned} F_N^{(m)}(z)&= \frac{1}{2^{m+1}} \sum _\alpha \Bigg [ \left( \frac{1 + p_\alpha }{2} + \frac{1 - p_\alpha }{2} \, z \right) ^{ N} \nonumber \\&\qquad \qquad \qquad + \left( \frac{1 - p_\alpha }{2} + \frac{1 + p_\alpha }{2} \, z \right) ^{ N} \, \Bigg ], \end{aligned}$$

with again $\lim _{m \rightarrow \infty } F_N^{(m)}(z) = F_N(z)$. The approximated equilibrium distribution is therefore an average over $2^{m+1}$ binomial distributions all with parameter *N* but with the $2^{m+1}$ probabilities ${\textstyle \frac{1}{2}}( 1 \pm \psi _0 \pm \cdots \pm \psi _m )$. Although it involves a sum over $2^{m+1}$ terms, it does not require high numerical precision to obtain stable results, and in general it provides a very efficient method for obtaining the equilibrium distribution for anything but very small *N*. The approximation can be made even more efficient by replacing a binomial distribution with parameter *N* and probability ${\textstyle \frac{1}{2}}(1 \pm p_\alpha )$ with a Gaussian distribution of mean ${\textstyle \frac{1}{2}}N (1 \pm p_\alpha )$ and variance $\frac{1}{4}N (1 - p_\alpha ^2)$ for *N* large enough.

In Fig. 13 we illustrate the complexity of the Hebb equilibrium distribution, for various choices of $\psi$ and *N*. As $\psi$ is reduced from unity to zero, the equilibrium distribution moves from bimodal via uniform to binomial (or Gaussian in the continuum limit). We focus on values of $\psi$ not far from $\psi = \frac{1}{2}$, for which the distribution in uniform, so as to capture this transition in the overall structure of the distribution, and also $\psi = 0.1$ and $\psi = 0.9$ at the extreme ranges. For fixed $\psi$, increasing *N* can create more oscillations in the distribution. The distribution respects the overall envelope for smaller *N*, but the maxima can split apart into multiple new maxima and minima as *N* increases. For fixed *N*, the oscillations spread out from the bimodal peaks at $i = 0$ and $i=N$ as $\psi$ is reduced, then they flip over in the transition through $\psi = \frac{1}{2}$, and finally they coalesce as $\psi$ is reduced to zero, where we expect the equilibrium distribution to become exactly binomial (or Gaussian). Even at $\psi = 0.1$, the equilibrium distribution is not far from Gaussian for larger values of *N*.

SNR lifetimes are not sensitive to the full equilibrium structure of the Hebb protocol. When obtaining SNR lifetimes from simulations, we therefore only need to ensure that the equilibrium distribution of synaptic strengths has the correct first- and second-order statistical structure. Defining $\underline{A}^\pm = \underline{A}_1 \pm \sqrt{\kappa _2} \, \underline{A}^\perp _1$, we find that

70a $$\begin{aligned} \underline{A}_1&= {\textstyle \frac{1}{2}}\left( \underline{A}^+ + \underline{A}^- \right) , \end{aligned}$$

70b $$\begin{aligned} \underline{A}_2&= {\textstyle \frac{1}{2}}\left( \underline{A}^+ \otimes \underline{A}^+ + \underline{A}^- \otimes \underline{A}^- \right) , \end{aligned}$$

70c $$\begin{aligned} \underline{A}_3&= {\textstyle \frac{1}{2}}\left( \underline{A}^+ \otimes \underline{A}^+ \otimes \underline{A}^+ + \underline{A}^- \otimes \underline{A}^- \otimes \underline{A}^- \right) . \end{aligned}$$

It is not possible in general to write $\underline{A}_N$ for $N \ge 4$ as a similar sum over tensor products with identical factors corresponding to probability distributions. However, the approximation

71 $$\begin{aligned} \underline{A}_N \approx {\textstyle \frac{1}{2}}\left( \underline{A}^+ \otimes \cdots \otimes \underline{A}^+ + \underline{A}^- \otimes \cdots \otimes \underline{A}^- \right) \end{aligned}$$

is exact for $N=1$, $N=2$ and $N=3$, and is guaranteed to have the correct marginal first-, second- and third-order statistical structure for $N \ge 4$. In simulations to obtain SNR lifetimes, it therefore suffices to prepare the equilibrium distribution according to Eq. (71) rather than the exact form in Eq. (56).

To compute $\tau _{\mathrm {mfpt}} = \langle \langle \tau _{\mathrm {mfpt}}(h_0 \, | \, N_{\mathrm {eff}}) \rangle _{h_0 > \vartheta } \rangle _{N_{\mathrm {eff}}}$ using the MIE approach in Eq. (4), we require the distribution of $h_0$ conditioned on exactly $N_{\mathrm {eff}}$ components of $\underline{\xi }^0$ being $+1$. For $\zeta = 0$, for which we can obtain $\mathsf {Prob}[ \, h \, | \, h' \,]$, the distribution of $h_0$ is equivalent to the distribution of the number of synapses of strength $+1$ after the storage of $\underline{\xi }^0$. Before the storage of $\underline{\xi }^0$, these $N_{\mathrm {eff}}$ synapses are in equilibrium, with probability $e_i(N_{\mathrm {eff}})$ that *i* of them have strength $+1$. During the storage of $\underline{\xi }^0$, the other $N_{\mathrm {eff}}-i$ may potentiate, each with probability *p*. The probability that *k* of these $N_{\mathrm {eff}}$ synapses have strength $+1$ after the storage of $\underline{\xi }^0$ is therefore just $\sum _{i=0}^k e_i(N_{\mathrm {eff}}) \, \Phi _{k-i}^{N_{\mathrm {eff}}- i}(p)$. Because of the convolution structure of this sum, we can find the PGF for these probabilities in terms of $F_{N_{\mathrm {eff}}}(z)$, obtaining (cf. Eq. (44))

72 $$\begin{aligned} F_{N_{\mathrm {eff}}}^+ (z) = \left[ (1-p) + p \, z \right] ^{N_{\mathrm {eff}}} F_{N_{\mathrm {eff}}} \left( \frac{z}{(1-p) + p \, z} \right) . \end{aligned}$$

Using $F_{N_{\mathrm {eff}}}^+ (z)$, we obtain the first two moments of $h_0$ as

73a $$\begin{aligned} \mathsf {E}[ \, h_0 \, | \, N_{\mathrm {eff}}\, ]&= \frac{N_{\mathrm {eff}}}{N} p, \end{aligned}$$

73b $$\begin{aligned} \mathsf {E}[ \, h_0^2 \, | \, N_{\mathrm {eff}}\, ]&= \frac{N_{\mathrm {eff}}}{N^2} + \frac{N_{\mathrm {eff}}(N_{\mathrm {eff}}-1)}{N^2} \left[ p^2 + \kappa _2 (1-p)^2 \right] , \end{aligned}$$

and by averaging over $N_{\mathrm {eff}}$, we recover $\mu (0)$ and $\sigma (0)^2$ in Eq. (7) (using Eq. (19a) for the correlation function) for $\zeta = 0$ and with $t=0$ s. For smaller values of $N_{\mathrm {eff}}$, we require the full conditional distribution of $h_0$ encoded in $F_{N_{\mathrm {eff}}}^+ (z)$, but for larger values, we can safely replace it by a Gaussian with the moments in Eq. (73).

## Footnotes

1 Strictly speaking we should work at discrete memory storage steps and only move to continuous time at the final step of the calculation, but as we drop covariance terms the difference is unimportant.

2 We stress that this condition is very different in character from the more usual condition that $\mu (0) / \sigma (0) > 1$, which is just the trivial requirement that the mean initial signal can be distinguished from $\mu (\infty ) \equiv 0$ at the one standard deviation level in models in which $\mu (t)$ falls monotonically.

## Declarations

### Code availability

Available upon request.
